# 2019 HRS/EHRA/APHRS/LAHRS expert consensus statement on catheter ablation of ventricular arrhythmias: Executive summary

**DOI:** 10.1016/j.hrthm.2019.03.014

**Published:** 2019-05-10

**Authors:** Edmond M. Cronin, Frank M. Bogun, Philippe Maury, Petr Peichl, Minglong Chen, Narayanan Namboodiri, Luis Aguinaga, Luiz Roberto Leite, Sana M. Al-Khatib, Elad Anter, Antonio Berruezo, David J. Callans, Mina K. Chung, Phillip Cuculich, Andre d’Avila, Barbara J. Deal, Paolo Della Bella, Thomas Deneke, Timm-Michael Dickfeld, Claudio Hadid, Haris M. Haqqani, G. Neal Kay, Rakesh Latchamsetty, Francis Marchlinski, John M. Miller, Akihiko Nogami, Akash R. Patel, Rajeev Kumar Pathak, Luis C. Saenz Morales, Pasquale Santangeli, John L. Sapp, Andrea Sarkozy, Kyoko Soejima, William G. Stevenson, Usha B. Tedrow, Wendy S. Tzou, Niraj Varma, Katja Zeppenfeld

**Affiliations:** 1 Hartford Hospital, Hartford, Connecticut; 2 University of Michigan, Ann Arbor, Michigan; 3 University Hospital Rangueil, Toulouse, France; 4 Institute for Clinical and Experimental Medicine, Prague, Czech Republic; 5 Jiangsu Province Hospital, The First Affiliated Hospital of Nanjing Medical University, Nanjing, China; 6 Sree Chitra Institute for Medical Sciences and Technology, Thiruvananthapuram, India; 7 Centro Privado de Cardiología, Tucuman, Argentina; 8 Instituto Brasília de Arritmia, Brasília, Brazil; 9 Duke University Medical Center, Durham, North Carolina; 10 Beth Israel Deaconess Medical Center, Boston, Massachusetts; 11 Heart Institute, Teknon Medical Center, Barcelona, Spain; 12 University of Pennsylvania, Philadelphia, Pennsylvania; 13 Cleveland Clinic, Cleveland, Ohio; 14 Washington University School of Medicine, St. Louis, Missouri; 15 Hospital Cardiologico SOS Cardio, Florianopolis, Brazil; 16 Northwestern University Feinberg School of Medicine, Chicago, Illinois; 17 Ospedale San Raffaele, Milan, Italy; 18 Herz- und Gefȧß-Klinik, Bad Neustadt, Germany; 19 University of Maryland, Baltimore, Maryland; 20 Hospital General de Agudos Cosme Argerich, Buenos Aires, Argentina; 21 University of Queensland, The Prince Charles Hospital, Chermside, Australia; 22 University of Alabama at Birmingham, Birmingham, Alabama; 23 Indiana University School of Medicine, Krannert Institute of Cardiology, Indianapolis, Indiana; 24 University of Tsukuba, Ibaraki, Japan; 25 University of California San Francisco Benioff Children’s Hospital, San Francisco, California; 26 Australian National University, Canberra Hospital, Canberra, Australia; 27 CardioInfantil Foundation, Cardiac Institute, Bogota, Columbia; 28 Queen Elizabeth II Health Sciences Centre, Halifax, Canada; 29 University Hospital Antwerp, University of Antwerp, Antwerp, Belgium; 30 Kyorin University School of Medicine, Tokyo, Japan; 31 Vanderbilt University Heart and Vascular Center, Nashville, Tennessee; 32 Brigham and Women’s Hospital, Boston, Massachusetts; 33 University of Colorado Denver, Aurora, Colorado; 34 Leiden University Medical Center, Leiden, the Netherlands.

**Keywords:** Catheter ablation, Clinical document, Electrical storm, Electroanatomical mapping, Electrocardiogram, Expert consensus statement, Imaging, Premature ventricular complex, Radiofrequency ablation, Ventricular arrhythmia, Ventricular tachycardia

## Abstract

Ventricular arrhythmias are an important cause of morbidity and mortality and come in a variety of forms, from single premature ventricular complexes to sustained ventricular tachycardia and fibrillation. Rapid developments have taken place over the past decade in our understanding of these arrhythmias and in our ability to diagnose and treat them. The field of catheter ablation has progressed with the development of new methods and tools, and with the publication of large clinical trials. Therefore, global cardiac electrophysiology professional societies undertook to outline recommendations and best practices for these procedures in a document that will update and replace the 2009 *EHRA/HRS Expert Consensus on Catheter Ablation of Ventricular Arrhythmias.* An expert writing group, after reviewing and discussing the literature, including a systematic review and meta-analysis published in conjunction with this document, and drawing on their own experience, drafted and voted on recommendations and summarized current knowledge and practice in the field. Each recommendation is presented in knowledge byte format and is accompanied by supportive text and references. Further sections provide a practical synopsis of the various techniques and of the specific ventricular arrhythmia sites and substrates encountered in the electrophysiology lab. The purpose of this document is to help electrophysiologists around the world to appropriately select patients for catheter ablation, to perform procedures in a safe and efficacious manner, and to provide follow-up and adjunctive care in order to obtain the best possible outcomes for patients with ventricular arrhythmias.

## 1 Introduction

### 1.1. Document Scope and Rationale

The field of electrophysiology has undergone rapid progress in the last decade, with advances both in our understanding of the genesis of ventricular arrhythmias (VAs) and in the technology used to treat them. In 2009, a joint task force of the European Heart Rhythm Association (EHRA) and the Heart Rhythm Society (HRS), in collaboration with the American College of Cardiology (ACC) and the American Heart Association (AHA), produced an expert consensus document that outlined the state of the field and defined the indications, techniques, and outcome measures of VA ablation (S1.1.1). In light of advances in the treatment of VAs in the interim, and the growth in the number of VA ablations performed in many countries and regions (S1.1.2,S1.1.3), an updated document is needed. This effort represents a worldwide partnership between transnational cardiac electrophysiology societies, namely, HRS, EHRA, the Asia Pacific Heart Rhythm Society (APHRS), and the Latin American Heart Rhythm Society (LAHRS), and collaboration with ACC, AHA, the Japanese Heart Rhythm Society (JHRS), the Brazilian Society of Cardiac Arrhythmias (Sociedade Brasileira de Arritmias Cardíacas [SOBRAC]), and the Pediatric and Congenital Electrophysiology Society (PACES). The consensus statement was also endorsed by the Canadian Heart Rhythm Society (CHRS).

This clinical document is intended to supplement, not replace, the *2017 AHA/ACC/HRS Guideline for Management of Patients with Ventricular Arrhythmias and the Prevention of Sudden Cardiac Death* (S1.1.4) and the *2015 ESC Guidelines for the Management of Patients with Ventricular Arrhythmias and the Prevention of Sudden Cardiac Death* (S1.1.5). The scope of the current document relates to ablation therapy for VAs, from premature ventricular complexes (PVCs) to monomorphic and polymorphic ventricular tachycardia (VT) and triggers of ventricular fibrillation (VF). Due to its narrower scope, the consensus statement delves into greater detail with regard to indications and technical aspects of VA ablation than the above-mentioned guidelines.

Where possible, the recommendations in this document are evidence based. It is intended to set reasonable standards that can be applicable worldwide, while recognizing the different resources, technological availability, disease prevalence, and health care delivery logistics in various parts of the world. In addition, parts of this document, particularly Section 9, present a practical guide on how to accomplish the procedures described in a manner that reflects the current standard of care, while recognizing that some procedures are better performed, and some disease states better managed, in settings in which there is specific expertise.

### 1.2. Methods

The writing group was selected according to each society’s procedures, including content and methodology experts representing the following organizations: HRS, EHRA, APHRS, LAHRS, ACC, AHA, JHRS, PACES, and SOBRAC. Each partner society nominated a chair and co-chair, who did not have relevant relationships with industry and other entities (RWIs). In accordance with HRS policies, disclosure of any RWIs was required from the writing committee members (Appendix 1) and from all peer reviewers (Appendix 2). Of the 38 committee members, 17 (45%) had no relevant RWIs. Recommendations were drafted by the members who did not have relevant RWIs. Members of the writing group conducted comprehensive literature searches of electronic databases, including Medline (via PubMed), Embase, and the Cochrane Library. Evidence tables were constructed to summarize the retrieved studies, with nonrandomized observational designs representing the predominant form of evidence (Appendix 3). Case reports were not used to support recommendations. Supportive text was drafted in the “knowledge byte” format for each recommendation. The writing committee discussed all recommendations and the evidence that informed them before voting. Initial failure to reach consensus was resolved by subsequent discussions, revisions as needed, and re-voting. Although the consensus threshold was set at 67%, all recommendations were approved by at least 80% of the writing committee members. The mean consensus over all recommendations was 95%. A quorum of two-thirds of the writing committee was met for all votes (S1.2.1).

Each recommendation in this document was assigned a Class of Recommendation (COR) and a Level of Evidence (LOE) according to the system developed by ACC and AHA (Table 1) (S1.2.2). The COR denotes the strength of the recommendation based on a careful assessment of the estimated benefits and risks; COR I indicates that the benefit of an intervention far exceeds its risk; COR IIa indicates that the benefit of the intervention moderately exceeds the risk; COR IIb indicates that the benefit may not exceed the risk; and COR III indicates that the benefit is equivalent to or is exceeded by the risk. The LOE reflects the quality of the evidence that supports the recommendation. LOE A is derived from high-quality randomized controlled trials; LOE B-R is derived from moderate-quality randomized controlled trials; LOE B-NR is derived from well-designed nonrandomized studies; LOE C-LD is derived from randomized or nonrandomized studies with limitations of design or execution; and LOE C-EO indicates that a recommendation was based on expert opinion (S1.2.2).

Unique to this consensus statement is the systematic review commissioned specifically for this document as part of HRS’s efforts to adopt the rigorous methodology required for guideline development. The systematic review was performed by an experienced evidence-based practice committee based at the University of Connecticut, which examined the question of VT ablation vs control in patients with VT and ischemic heart disease (IHD) (S1.2.3). The question, in PICOT format, was as follows: In adults with history of sustained VT and IHD, what is the effectiveness and what are the detriments of catheter ablation compared with other interventions? Components of the PICOT were as follows: P = adults with history of sustained VT and IHD; I = catheter ablation; C = control (no therapy or antiarrhythmic drug [AAD]); O = outcomes of interest, which included 1) appropriate implantable cardioverter defibrillator (ICD) therapies (ICD shock or antitachycardia pacing [ATP]), 2) appropriate ICD shocks, 3) VT storm (defined as three shocks within 24 hours), 4) recurrent VT/VF, 5) cardiac hospitalizations, and 6) all-cause mortality; and T = no time restrictions.

An industry forum was conducted to achieve a structured dialogue to address technical questions and to gain a better understanding of future directions and challenges. Because of the potential for actual or perceived bias, HRS imposes strict parameters on information sharing to ensure that industry participates only in an advisory capacity and has no role in either the writing of the document or its review.

The draft document underwent review by the HRS Scientific and Clinical Documents Committee and was approved by the writing committee. Recommendations were subject to a period of public comment, and the entire document underwent rigorous peer review by each of the participating societies and revision by the Chairs, before endorsement.

## 2 Background

**Figure 1:**
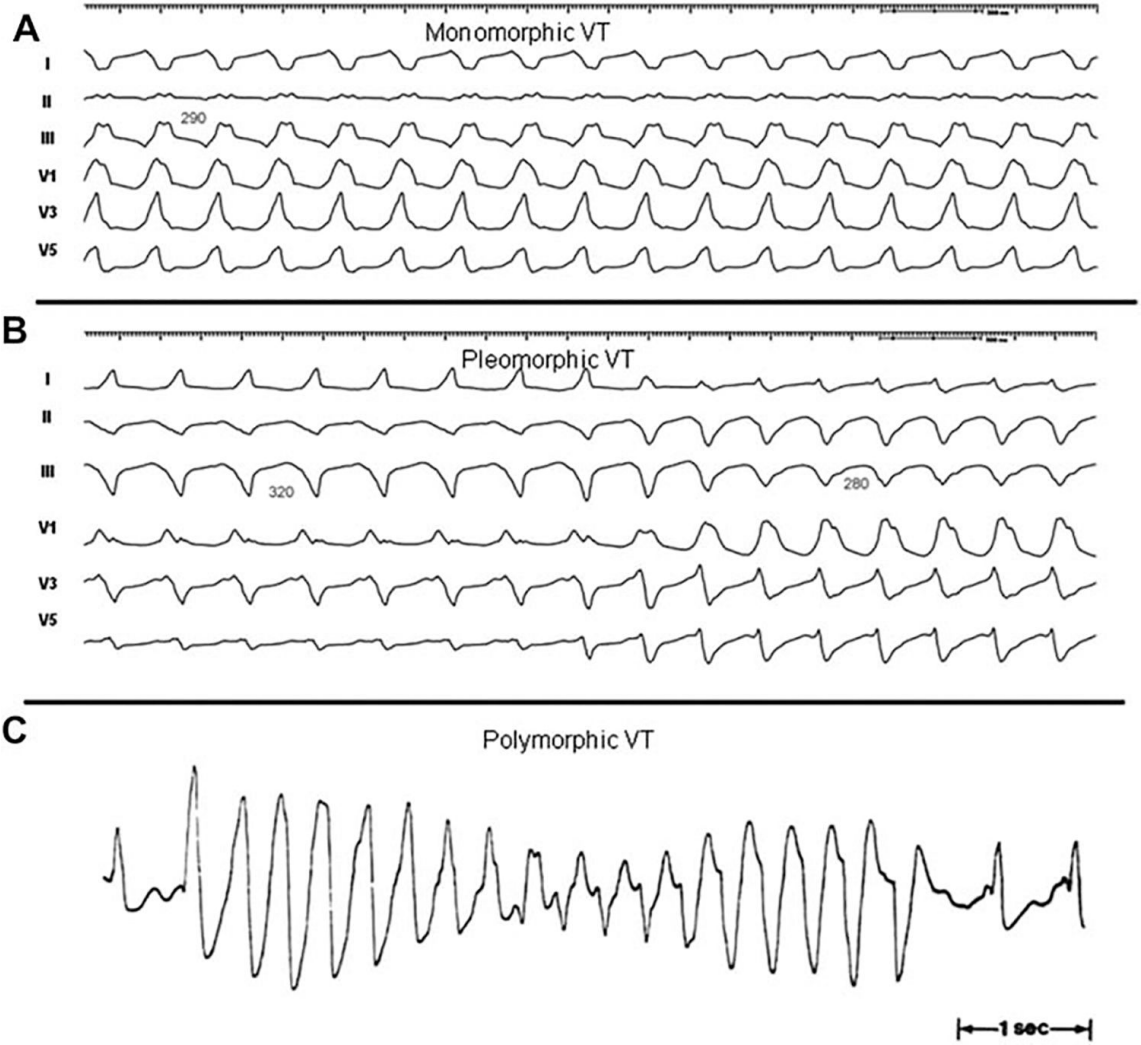
Monomorphic (A), pleomorphic (B), and polymorphic (C) VT. Reproduced with permission of the Heart Rhythm Society from Aliot et al. EHRA/HRS expert consensus on catheter ablation of ventricular arrhythmias. Heart Rhythm 2009;6:886–933. VT = ventricular tachycardia.

This section reviews the history of VT ablation, details the mechanisms of VT, and provides definitions of frequently used terms (Table 2), including anatomic definitions (Table 3), as well as illustrating some types of sustained VA (Figure 1).

## 3 Clinical Evaluation

This section discusses clinical presentations of patients with VAs and their workup as it pertains to documentation of arrhythmias and appropriate testing to assess for the presence of SHD.

### 3.1. Clinical Presentation

**Table:** Recommendation for clinical evaluation of patients with VAs

COR	LOE	Recommendation

I	C-EO	1. A careful clinical evaluation including history, physical examination, review of available cardiac rhythm data, prior imaging, and relevant laboratory workup should be performed in patients presenting with VAs.

### 3.2. Diagnostic Evaluation

#### 3.2.1. Resting 12-Lead Electrocardiogram

**Table:** Recommendations for resting 12-lead ECG

COR	LOE	Recommendations	References

**I**	**B-NR**	**1. In patients with wide complex tachycardia, a 12-lead ECG during tachycardia should be obtained whenever possible.**	S3.2.1.1–S3.2.1.15
**I**	**B-NR**	**2. In patients with suspected or documented VA, a 12-lead ECG should be obtained in sinus rhythm to look for evidence of underlying heart disease.**	S3.2.1.16

#### 3.2.2. Assessment of Structural Heart Disease and Myocardial Ischemia

**Table:** Recommendations for assessment of SHD and myocardial ischemia

COR	LOE	Recommendations	References

**I**	**B-NR**	**1. In patients with known or suspected VA, echocardiography is recommended for evaluation of cardiac structure and function.**	S3.2.2.1,S3.2.2.2
**IIa**	**B-NR**	**2. In patients presenting with VA who are suspected of having SHD, even after normal echocardiographic evaluation, advanced cardiac imaging can be useful to detect and characterize underlying SHD.**	S3.2.2.3–S3.2.2.7
**IIa**	**C-EO**	**3. In patients with VA in whom myocardial ischemia is suspected, stress testing and/or coronary angiography and subsequent revascularization can be beneficial before catheter ablation to avoid significant ischemia during induced VT.**	
**III: No Benefit**	**B-NR**	**4. In patients presenting with monomorphic VT, revascularization alone is not effective to prevent VT recurrence.**	S3.2.2.8–S3.2.2.10

#### 3.2.3. Risk Stratification in the Setting of Frequent Premature Ventricular Complexes

**Table:** Recommendations for cardiac magnetic resonance imaging (CMR) in patients with frequent PVCs and for PES in patients with SHD and frequent PVCs

COR	LOE	Recommendations	References

**IIa**	**B-NR**	**1. CMR can be useful for risk stratification for sudden cardiac death in patients with frequent PVCs.**	S3.2.3.1,S3.2.3.2
**IIa**	**C-LD**	**2. PES can be useful for risk stratification for sudden cardiac death in patients with SHD undergoing ablation of frequent PVCs.**	S3.2.3.2

#### 3.2.4. Longitudinal Follow-up in the Setting of Frequent Premature Ventricular Complexes

**Table:** Recommendation for longitudinal follow-up of patients with frequent PVCs

COR	LOE	Recommendation	Reference

**IIa**	**B-NR**	**1. Periodic monitoring of PVC burden and LV function and dimensions can be useful in patients with frequent, asymptomatic PVCs and normal LV function and dimensions.**	S3.2.4.1

## 4 Indications for Catheter Ablation

Following are the consensus recommendations for catheter ablation of VAs organized by underlying diagnosis and substrate. These recommendations are each assigned a COR and an LOE according to the current recommendation classification system (S4.1). In drafting each of these recommendations, the writing committee took into account the published literature in the specific area, including the methodological quality and size of each study, as well as the collective clinical experience of the writing group when published data were not available. Implicit in each recommendation are several points: 1) the procedure is being performed by an electrophysiologist with appropriate training and experience in the procedure and in a facility with appropriate resources; 2) patient and procedural complexity vary widely, and some patients or situations merit a more experienced operator or a center with more capabilities than others, even within the same recommendation (eg, when an epicardial procedure is indicated and the operator or institution has limited experience with this procedure, it might be preferable to refer the patient to an operator or institution with adequate experience in performing epicardial procedures); 3) the patient is an appropriate candidate for the procedure, as outlined in Section 5, recognizing that the level of patient suitability for a procedure will vary widely with the clinical scenario; and 4) the patient’s (or designee’s) informed consent, values, and overall clinical trajectory are fundamental to a decision to proceed (or not) with any procedure. Therefore, in some clinical scenarios, initiation or continuation of medical therapy instead of an ablation procedure may be the most appropriate option, even when a class 1 recommendation for ablation is present. There may also be scenarios not explicitly covered in this document, and on which little or no published literature is available, in which the physician and patient must rely solely on their own judgment.

**Figure 2:**
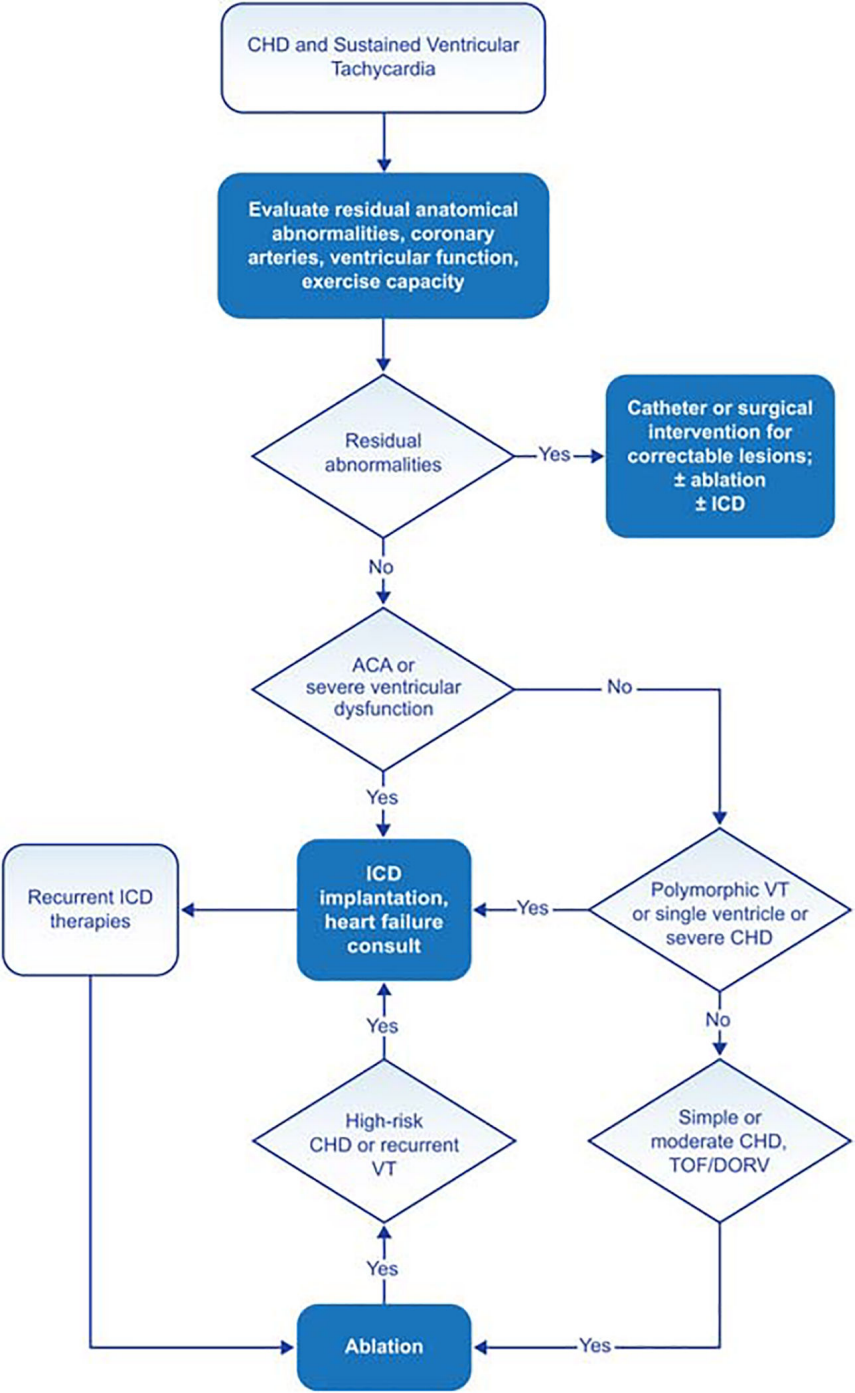
Congenital heart disease and sustained VT. For further discussion of ICD candidacy, please see *PACES/HRS Expert Consensus Statement on the Recognition and Management of Arrhythmias in Adult Congenital Heart Disease* (S4.7.14) and *2012 ACCF/AHA/HRS Focused Update of the 2008 Guidelines for Device-Based Therapy of Cardiac Rhythm Abnormalities* (S4.7.26). ACA = aborted cardiac arrest; CHD = congenital heart disease; DORV = double outlet right ventricle; ICD = implantable cardioverter defibrillator; TOF = tetralogy of Fallot; VT = ventricular tachycardia.

Figure 2 provides an overview of care for the patient with congenital heart disease (CHD) and VA.

### 4.1. Idiopathic Outflow Tract Ventricular Arrhythmia

**Table:** Recommendations for catheter ablation of idiopathic outflow tract VA

COR	LOE	Recommendations	References

**I**	**B-R**	**1. In patients with frequent and symptomatic PVCs originating from the RVOT in an otherwise normal heart, catheter ablation is recommended in preference to metoprolol or propafenone.**	S4.1.1
**I**	**B-NR**	**2. In patients with symptomatic VAs from the RVOT in an otherwise normal heart for whom antiarrhythmic medications are ineffective, not tolerated, or not the patient’s preference, catheter ablation is useful.**	S4.1.2–S4.1.12
**I**	**B-NR**	**3. In patients with symptomatic idiopathic sustained monomorphic VT, catheter ablation is useful.**	S4.1.13–S4.1.17
**IIa**	**B-NR**	**4. In patients with symptomatic VAs from the endocardial LVOT, including the SV, in an otherwise normal heart for whom antiarrhythmic medications are ineffective, not tolerated, or not the patient’s preference, catheter ablation can be useful.**	S4.1.18–S4.1.27
**IIa**	**B-NR**	**5. In patients with symptomatic VAs from the epicardial outflow tract or LV summit in an otherwise normal heart for whom antiarrhythmic medications are ineffective, not tolerated, or not the patient’s preference, catheter ablation can be useful.**	S4.1.28–S4.1.32

### 4.2. Idiopathic Nonoutflow Tract Ventricular Arrhythmia

**Table:** Recommendations for catheter ablation of nonoutflow tract VAs in the absence of SHD

COR	LOE	Recommendations	References

**I**	**B-NR**	**1. In patients with symptomatic VAs from the RV at sites other than the outflow tracts (tricuspid annulus, moderator band, parietal band, or papillary muscles) in an otherwise normal heart for whom antiarrhythmic medications are ineffective, not tolerated, or not the patient’s preference, catheter ablation is useful.**	S4.2.1–S4.2.14
**I**	**B-NR**	**2. In patients with symptomatic VAs from the LV at sites other than the outflow tracts (mitral annulus, papillary muscles, or AMC) in an otherwise normal heart for whom antiarrhythmic medications are ineffective, not tolerated, or not the patient’s preference, catheter ablation is useful.**	S4.2.15–S4.2.31
**IIa**	**B-NR**	**3. In patients with symptomatic VAs from the epicardial coronary venous system in an otherwise normal heart for whom antiarrhythmic medications are ineffective, not tolerated, or not the patient’s preference, catheter ablation can be useful.**	S4.2.32–S4.2.43
**IIa**	**B-NR**	**4. In patients with symptomatic VAs from para-Hisian sites in an otherwise normal heart for whom antiarrhythmic medications are ineffective, not tolerated, or not the patient’s preference, catheter ablation can be useful.**	S4.2.7,S4.2.13,S4.2.14,S4.2.44–S4.2.49
**IIa**	**C-LD**	**5. In patients with symptomatic VAs from the posterior-superior process of the LV in an otherwise normal heart for whom antiarrhythmic medications are ineffective, not tolerated, or not the patient’s preference, catheter ablation from the LV endocardium, right atrium, or CS, can be useful.**	S4.2.50–S4.2.52

### 4.3. Premature Ventricular Complexes With or Without Left Ventricular Dysfunction

**Table:** Recommendations for catheter ablation of PVCs in patients with or without LV dysfunction

COR	LOE	Recommendations	References

**I**	**B-NR**	**1. In patients with cardiomyopathy suspected to be caused by frequent and predominately monomorphic PVCs and for whom AADs are ineffective, not tolerated, or not preferred for long-term therapy, catheter ablation is recommended.**	S4.3.1–S4.3.10
**IIa**	**B-NR**	**2. In patients with SHD in whom frequent PVCs are suspected to be contributing to a cardiomyopathy and for whom AADs are ineffective, not tolerated, or not preferred for long-term therapy, catheter ablation can be useful.**	S4.3.3,S4.3.11,S4.3.12
**IIa**	**B-NR**	**3. In patients with focally triggered VF refractory to AADs and triggered by a similar PVC, catheter ablation can be useful.**	S4.3.13–S4.3.17
**IIa**	**C-LD**	**4. In nonresponders to cardiac resynchronization therapy with very frequent unifocal PVCs limiting optimal biventricular pacing despite pharmacological therapy, catheter ablation can be useful.**	S4.3.18

### 4.4. Ventricular Arrhythmia in Ischemic Heart Disease

**Table:** Recommendations for catheter ablation of VAs in patients with IHD

COR	LOE	Recommendations	References

**I**	**B-R**	**1. In patients with IHD who experience recurrent monomorphic VT despite chronic amiodarone therapy, catheter ablation is recommended in preference to escalating AAD therapy.**	S4.4.1
**I**	**B-NR**	**2. In patients with IHD and recurrent symptomatic monomorphic VT despite AAD therapy, or when AAD therapy is contraindicated or not tolerated, catheter ablation is recommended to reduce recurrent VT.**	S4.4.2–S4.4.4
**I**	**B-NR**	**3. In patients with IHD and VT storm refractory to AAD therapy, catheter ablation is recommended.**	S4.4.5–S4.4.9
**IIa**	**C-EO**	**4. In patients with IHD and recurrent monomorphic VT, in whom AADs are not desired, catheter ablation can be useful.**	
**IIb**	**A**	**5. In patients with IHD and an ICD who experience a first episode of monomorphic VT, catheter ablation may be considered to reduce the risk of recurrent VT or ICD therapies.**	S4.4.10–S4.4.14
**IIb**	**C-LD**	**6. In patients with prior myocardial infarction and recurrent episodes of symptomatic sustained VT for whom prior endocardial catheter ablation has not been successful and who have ECG, endocardial mapping, or imaging evidence of a subepicardial VT substrate, epicardial ablation may be considered.**	S4.4.15–S4.4.19

### 4.5. Nonischemic Cardiomyopathy

**Table:** Recommendations for catheter ablation of VT in nonischemic cardiomyopathy (NICM)

COR	LOE	Recommendations	References

**I**	**B-NR**	**1. In patients with NICM and recurrent sustained monomorphic VT for whom antiarrhythmic medications are ineffective, contraindicated, or not tolerated, catheter ablation is useful for reducing recurrent VT and ICD shocks.**	S4.5.1–S4.5.6
**I**	**B-NR**	**2. In patients with NICM and electrical storm refractory to AAD therapy, catheter ablation is useful for reducing recurrent VT and ICD shocks.**	S4.5.7–S4.5.9
**IIa**	**B-NR**	**3. In patients with NICM, epicardial catheter ablation of VT can be useful after failure of endocardial ablation or as the initial ablation approach when there is a suspicion of an epicardial substrate or circuit.**	S4.5.4,S4.5.10–S4.5.13
**IIa**	**B-NR**	**4. In patients with cardiac sarcoidosis and recurrent VT despite medical therapy, catheter ablation can be useful to reduce the risk of VT recurrence and ICD shocks.**	S4.5.14–S4.5.18
**IIa**	**C-EO**	**5. In patients with NICM and recurrent sustained monomorphic VT for whom antiarrhythmic medications are not desired, catheter ablation can be useful for reducing recurrent VT and ICD shocks.**	
**IIb**	**B-NR**	**6. In patients with NICM related to lamin A/C (*LMNA*) mutations and recurrent VT, catheter ablation may be considered as a palliative strategy for short-term arrhythmia control.**	S4.5.19

### 4.6. Ventricular Arrhythmia Involving the His-Purkinje System, Bundle Branch Reentrant Ventricular Tachycardia, and Fascicular Ventricular Tachycardia

**Table:** Recommendations for catheter ablation of bundle branch reentrant VT and for catheter ablation of fascicular VT

COR	LOE	Recommendations	References

**I**	**B-NR**	**1. In patients with bundle branch reentrant VT, catheter ablation is useful for reducing the risk of recurrent VT.**	S4.6.1–S4.6.9
**I**	**B-NR**	**2. In patients with idiopathic left fascicular reentrant VT for whom medications are ineffective, not tolerated, or not the patient’s preference, catheter ablation is useful.**	S4.6.10–S4.6.22
**I**	**B-NR**	**3. In larger pediatric patients (≥15 kg) with idiopathic left fascicular reentrant VT in whom medical treatment is ineffective or not tolerated, catheter ablation is useful.**	S4.6.23–S4.6.26
**I**	**B-NR**	**4. In patients with focal fascicular VT with or without SHD, catheter ablation is useful.**	S4.6.11,S4.6.27–S4.6.29
**I**	**B-NR**	**5. In patients with postinfarction reentrant Purkinje fiber-mediated VT, catheter ablation is useful.**	S4.6.30–S4.6.32

### 4.7. Congenital Heart Disease

**Table:** Recommendations for catheter ablation of VA in patients with CHD

COR	LOE	Recommendations	References

**I**	**B-NR**	**1. In patients with CHD presenting with sustained VAs, evaluation for potential residual anatomical or coronary abnormalities should be performed.**	S4.7.1–S4.7.6
**I**	**B-NR**	**2. In patients with CHD presenting with sustained VT in the presence of important hemodynamic lesions, treatment of hemodynamic abnormalities as feasible should be performed in conjunction with consideration for ablation.**	S4.7.2,S4.7.7–S4.7.16
**I**	**B-NR**	**3. In patients with repaired tetralogy of Fallot and sustained monomorphic VT or recurrent appropriate ICD therapy for VAs, catheter ablation is effective.**	S4.7.17–S4.7.24
**IIa**	**B-NR**	**4. In select patients with CHD and clinical episodes of sustained VT who are undergoing surgical repair of residual hemodynamic abnormalities, surgical ablation of VT guided by preoperative or intraoperative electroanatomical mapping (EAM) can be beneficial.**	S4.7.2,S4.7.8,S4.7.9,S4.7.11,S4.7.25

### 4.8. Inherited Arrhythmia Syndromes

**Table:** Recommendations for catheter ablation of VA in inherited primary arrhythmia disorders

COR	LOE	Recommendations	References

**I**	**B-NR**	**1. In patients with arrhythmogenic right ventricular cardiomyopathy (ARVC) who experience recurrent sustained VT or frequent appropriate ICD interventions for VT in whom AAD therapy is ineffective or not tolerated, catheter ablation, at a center with specific expertise, is recommended.**	S4.8.1–S4.8.11
**I**	**B-NR**	**2. In patients with ARVC who have failed one or more attempts of endocardial VT catheter ablation, an epicardial approach for VT ablation is recommended.**	S4.8.3–S4.8.7,S4.8.12,S4.8.13
**IIa**	**B-NR**	**3. In patients with ARVC who experience recurrent sustained VT or frequent appropriate ICD interventions for VT in whom AAD therapy is not desired or preferred, catheter ablation, at a center with specific expertise, is reasonable.**	S4.8.1,S4.8.3–S4.8.6,S4.8.8
**IIa**	**B-NR**	**4. In patients with Brugada syndrome who experience recurrent sustained VAs or frequent appropriate ICD interventions, catheter ablation can be useful.**	S4.8.14–S4.8.17
**IIa**	**C-LD**	**5. In patients with ARVC, a first-line combined endocardial/epicardial approach for VT ablation is reasonable.**	S4.8.1,S4.8.6,S4.8.12,S4.8.18

### 4.9. Ventricular Arrhythmia in Hypertrophic Cardiomyopathy

**Table:** Recommendation for VA ablation in hypertrophic cardiomyopathy (HCM)

COR	LOE	Recommendation	References

**IIa**	**B-NR**	**1. In patients with HCM and recurrent monomorphic VT in whom AAD therapy is ineffective or not tolerated, catheter ablation can be useful.**	S4.9.1–S4.9.5

## 5 Procedural Planning

**Figure 3:**
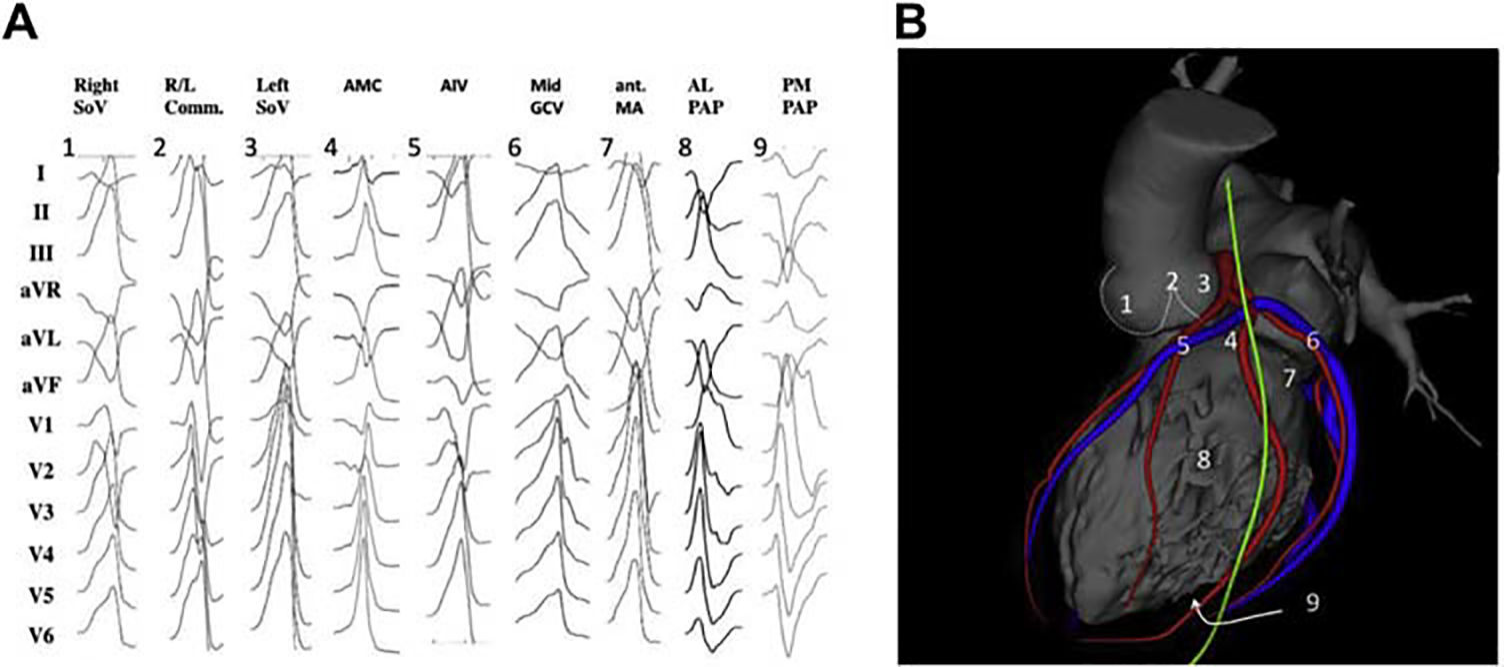
Examples of 12-lead ECGs of premature ventricular complexes from different LV sites, as corroborated by successful focal ablation. (A) shows 12-lead ECG patterns of common ventricular arrhythmia origins in patients without SHD [1–9] from the left ventricle. All leads are displayed at the same amplification and sweep speed. These locations are illustrated in (B) based on 3D reconstruction of a cardiac computed tomography using the MUSIC software that was developed at the University of Bordeaux. The reconstruction shows an anterolateral view of the left ventricle, aorta, and left atrium. Also shown are the coronary arteries (red), the coronary venous system (blue), and the phrenic nerve (green). AIV = anterior interventricular vein; AL PAP = anterolateral papillary muscle; AMC = aortomitral continuity; ECG = electrocardiogram; GCV = great cardiac vein; ant. MA = anterior mitral valve annulus; PM PAP = posteromedial papillary muscle; R/L = right-left; SHD = structural heart disease; SoV = sinus of Valsalva.

**Figure 4:**
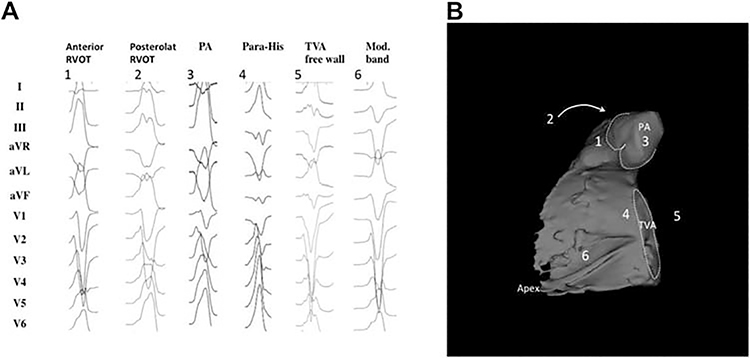
Examples of 12-lead ECGs of premature ventricular complexes from different right ventricular sites, as corroborated by successful focal ablation. All leads are displayed at the same amplification and sweep speed. (A) shows the 12-lead ECG pattern of common origins of right ventricular arrhythmias in patients without SHD [1–6]. The locations are detailed in a 3D reconstruction of the computed tomography using the MUSIC software that was developed at the University of Bordeaux. The reconstruction shown in (B) illustrates the septal view of the right ventricle. Indicated are the pulmonary artery, the tricuspid valve annulus, and the right ventricular apex. ECG = electrocardiogram; PA = pulmonary artery; RVOT= right ventricular outflow tract; SHD = structural heart disease; TVA = tricuspid valve annulus.

This section includes preprocedural risk assessment (Table 4), preprocedural patient preparation, and preprocedural arrhythmia documentation with a focus on the regionalizing information of the ECG regarding the origin of VAs (Figures 3 and 4). Furthermore, the capabilities of multimodality imaging in localizing the arrhythmogenic substrate are discussed in detail. Topics including the required equipment, personnel, and facility are detailed in this section.

**Table:** Recommendations for preprocedural imaging for VA catheter ablation

COR	LOE	Recommendations	References

**I**	**B-NR**	**1. In patients with LV dysfunction undergoing catheter ablation of VA, preprocedural or intraprocedural imaging is recommended to rule out cardiac thrombi.**	S5.1–S5.6
**IIa**	**B-NR**	**2. In patients with NICM or ischemic cardiomyopathy (ICM) undergoing catheter ablation of VT, preprocedural CMR can be useful to reduce VT recurrence.**	S5.7–S5.9
**IIa**	**B-NR**	**3. In patients with NICM or ICM undergoing catheter ablation of VA, preprocedural imaging can be useful for procedural planning.**	S5.10–S5.26
**IIa**	**C-EO**	**4. In patients with NICM, CMR can be useful prior to ICD implantation to allow imaging without device-related artifact for diagnostic purposes and identification of potential arrhythmogenic substrate.**	
**IIb**	**C-EO**	**5. In patients with ICM, CMR may be considered prior to ICD implantation to allow imaging without device-related artifact for identification of the potential arrhythmogenic substrate.**	

## 6 Intraprocedural Patient Care

Important aspects regarding intraprocedural sedation and its potential problems are highlighted in this section. Furthermore, vascular access, epicardial access with its many potential complications are discussed in detail, as well as anticoagulation and the indications for the use of hemodynamic support (HS) during VT ablation procedures.

### 6.1. Anesthesia

**Table:** Recommendations for anesthesia during catheter ablation of VA

COR	LOE	Recommendations	References

**I**	**C-EO**	**1. Provision of variable depth of sedation, analgesia, and anesthesia during mapping and ablation of VA is recommended.**	
**I**	**C-EO**	**2. In patients undergoing VA ablation, careful preprocedural assessment is indicated to define the ideal strategy for sedation and analgesia.**	
**IIa**	**C-LD**	**3. It is reasonable to avoid general anesthesia and deeper levels of sedation in patients with idiopathic VA, particularly if the arrhythmia is suspected to be catecholamine-sensitive or was not inducible at a prior procedure.**	S6.1.1
**IIb**	**B-NR**	**4. Moderate to deep sedation under close hemodynamic and respiratory monitoring might be considered for VA ablation in stable patients with idiopathic or scar-related VAs expected to have a longer procedure or undergo a painful technique, such as epicardial access.**	S6.1.1–S6.1.3

### 6.2. Vascular Access

**Table:** Recommendation for vascular access during catheter ablation of VA

COR	LOE	Recommendation	References

**I**	**B-NR**	**1. Ultrasound-guided femoral arterial and venous access is recommended to reduce the incidence of vascular access complications during VA ablation.**	S6.2.1–S6.2.5

### 6.3. Epicardial Access

**Table:** Recommendations for epicardial access for catheter ablation

COR	LOE	Recommendations

**I**	**C-EO**	**1. In patients undergoing epicardial VT ablation, imaging of the epicardial coronary arteries by coronary arteriography or coronary computed tomography angiogram prior to ablation is recommended to reduce the risk of arterial injury.**
**I**	**C-EO**	**2. In patients undergoing epicardial VT ablation via a percutaneous approach, provision for immediate echocardiography, blood transfusion, and onsite cardiothoracic surgical backup is recommended.**
**I**	**C-EO**	**3. In patients with prior cardiac surgery or pericardial adhesions for whom epicardial VT ablation via a percutaneous approach is considered, careful assessment of the risk/benefit ratio and alternative therapies such as surgical dissection are recommended.**
**I**	**C-EO**	**4. In patients undergoing epicardial VT ablation, pacing with high stimulus intensity from the ablation electrode to rule out diaphragmatic stimulation is recommended to avoid phrenic nerve injury.**

### 6.4. Intraprocedural Hemodynamic Support

**Table:** Recommendations for catheter ablation of VA with mechanical HS

COR	LOE	Recommendations	References

**I**	**C-EO**	**1. In select patients at risk of requiring HS, a decision to proceed with catheter ablation of VA should be made in collaboration with specialists in advanced heart failure management.**	
**IIa**	**B-NR**	**2. In select patients, HS with a percutaneous ventricular assist device and extracorporeal membrane oxygenation during VT ablation can be useful to avoid acute hemodynamic deterioration.**	S6.4.1–S6.4.7
**IIb**	**B-NR**	**3. Mechanical HS may be considered in select cases to allow mapping and ablation of unstable VTs.**	S6.4.1–S6.4.6

### 6.5. Intraprocedural Anticoagulation

**Table:** Recommendations for intraprocedural anticoagulation

COR	LOE	Recommendations	References

**I**	**B-NR**	**1. In patients undergoing endocardial LV catheter mapping and/or ablation, intraprocedural systemic anticoagulation with intravenous heparin is recommended.**	S6.5.1–S6.5.6
**I**	**C-EO**	**2. In patients undergoing RV endocardial mapping and/or ablation who are considered high risk for thromboembolism, intraprocedural systemic anticoagulation with intravenous heparin is recommended.**	
**IIa**	**C-LD**	**3. In patients undergoing epicardial access after systemic heparinization, reversal of heparin with protamine is reasonable.**	S6.5.7,S6.5.8

## 7 Electrophysiological Testing

The benefits and limitations of PES are detailed in this section.

## 8 Mapping and Imaging Techniques

### 8.1. Overview

**Figure 5:**
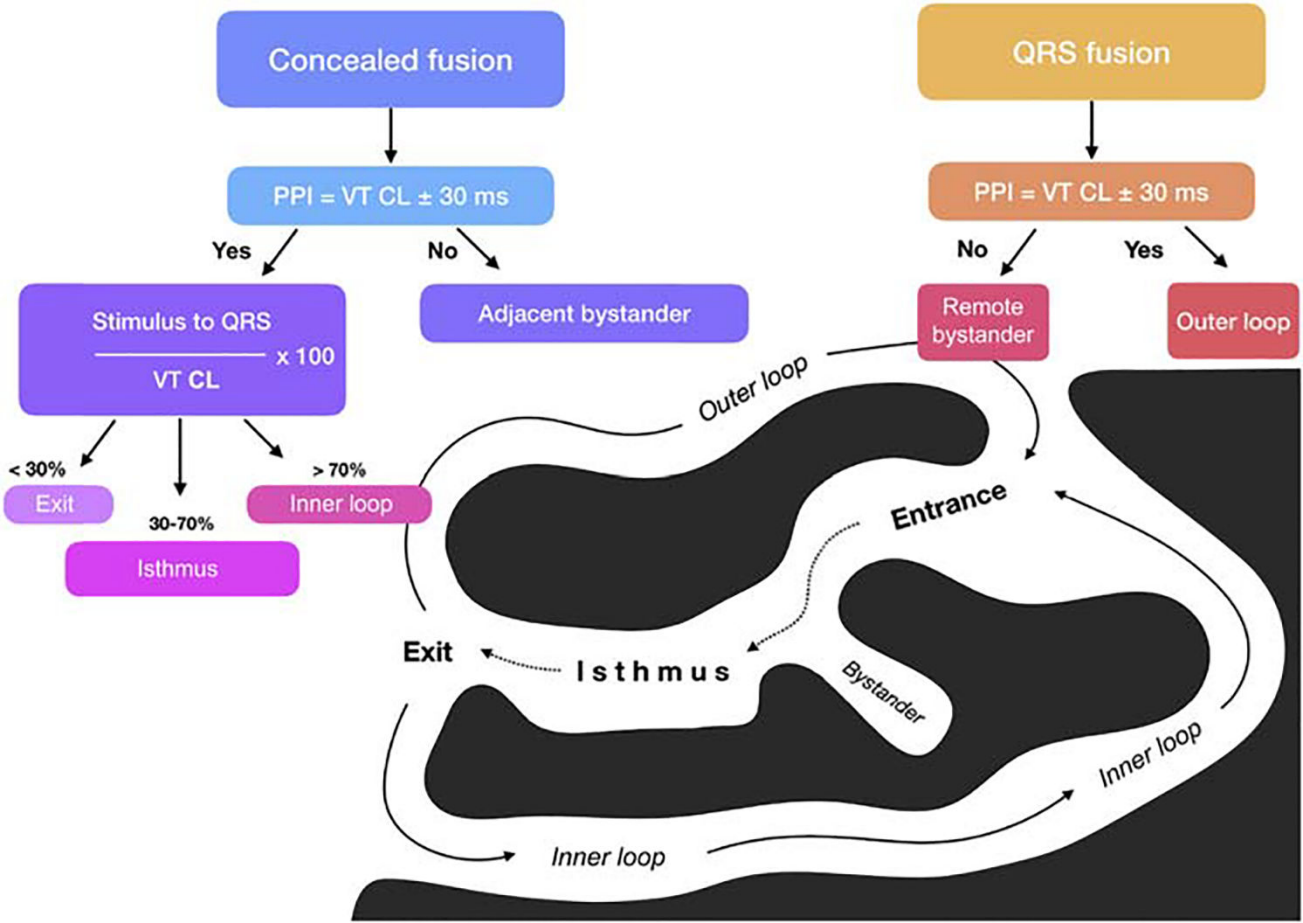
Entrainment responses from components of reentrant VT circuit. CL = cycle length; PPI = postpacing interval; VT = ventricular tachycardia. Adapted with permission from Elsevier (Stevenson et al. J Am Coll Cardiol 1997;29:1180–1189).

**Figure 6:**
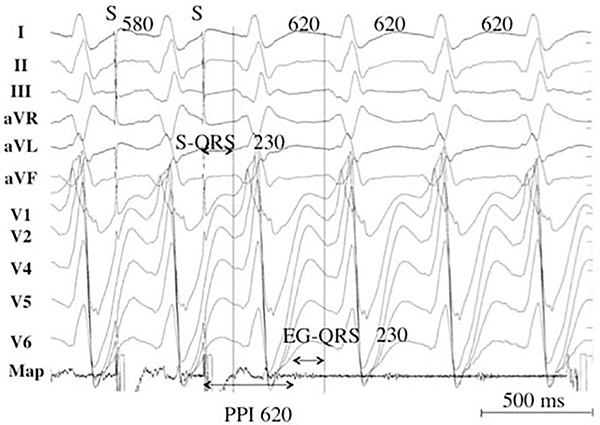
Pacing from the protected isthmus of a VT circuit. Entrainment mapping during VT. The VT CL is 620 ms, and pacing is performed at a CL of 580 ms. A low-voltage electrogram is located in diastole on the recordings of the ablation catheter (Map). The stimulus-QRS interval is 230 ms and matches with the electrogram-QRS interval. The postpacing interval is equal to the VT CL. The stimulus-QRS/VT CL ratio is 0.37, indicating that the catheter is located in the common pathway. CL = cycle length; PPI = postpacing interval; VT = ventricular tachycardia.

Activation mapping with multipolar catheters, entrainment mapping (Figures 5 and 6), and pace mapping are the main techniques used to map VAs. This section reviews these techniques including the technique of substrate mapping aiming to identify the arrhythmogenic substrate in sinus rhythm. Furthermore, intraprocedural imaging as it pertains to procedural safety and to identification of the arrhythmogenic substrate is reviewed in this section.

### 8.2. Substrate Mapping in Sinus Rhythm

**Table:** Recommendations for substrate mapping in sinus rhythm

COR	LOE	Recommendations	References

**I**	**B-NR**	**1. In patients with scar-related VT, substrate-guided ablation is useful for prevention of arrhythmia recurrences.**	S8.2.1–S8.2.11
**IIa**	**B-NR**	**2. High-density multielectrode mapping to obtain a more comprehensive characterization of the arrhythmogenic tissue during catheter ablation of scar-related VT can be useful.**	S8.2.12–S8.2.14
**IIa**	**B-NR**	**3. In patients with no or minimal endocardial bipolar electrogram abnormalities, reduced unipolar voltage can be useful for detection of epicardial or intramural scar.**	S8.2.15–S8.2.19

### 8.3. Intraprocedural Imaging During Catheter Ablation of Ventricular Arrhythmias

**Table:** Recommendations for intraprocedural imaging during catheter ablation of VAs

COR	LOE	Recommendations	References

**I**	**B-NR**	**1. Coronary angiography or intracardiac echocardiography (ICE) is recommended to localize the ostia of the coronary arteries prior to ablation within the SV.**	S8.3.1–S8.3.4
**I**	**B-NR**	**2. Coronary angiography is recommended to identify the course of the coronary arteries when ablation is performed in the coronary venous system or in the epicardium.**	S8.3.5–S8.3.8
**I**	**B-NR**	**3. ICE is beneficial to identify and target the papillary muscles with ablation and to assess for catheter stability.**	S8.3.9–S8.3.20
**I**	**B-NR**	**4. ICE or transthoracic echocardiography is useful to assess for pericardial effusion in case of hemodynamic deterioration of the patient.**	S8.3.21–S8.3.23
**I**	**C-LD**	**5. ICE is useful for early recognition of complications, including pericardial effusion.**	S8.3.21–S8.3.23
**IIb**	**B-NR**	**6. ICE may be useful as an adjuvant technique to identify wall segments with wall thinning, wall motion abnormalities, and segments with increased echogenicity, and also to identify intracardiac thrombi.**	S8.3.24,S8.3.25

### 8.4. Electroanatomical Mapping Systems and Robotic Navigation

**Table:** Recommendations for the use of EAM systems and remote navigation in ablation procedures for VAs

COR	LOE	Recommendations	References

**I**	**B-NR**	**1. In patients with VA due to SHD undergoing an ablation procedure, EAM is useful.**	S8.4.1–S8.4.9
**IIa**	**B-NR**	**2. In patients with idiopathic VA undergoing an ablation procedure, EAM can be useful.**	S8.4.4,S8.4.6
**IIa**	**B-NR**	**3. In patients undergoing an ablation procedure for VA, magnetic catheter navigation can be useful to reduce fluoroscopy use.**	S8.4.10–S8.4.14

## 9 Mapping and Ablation

**Figure 7:**
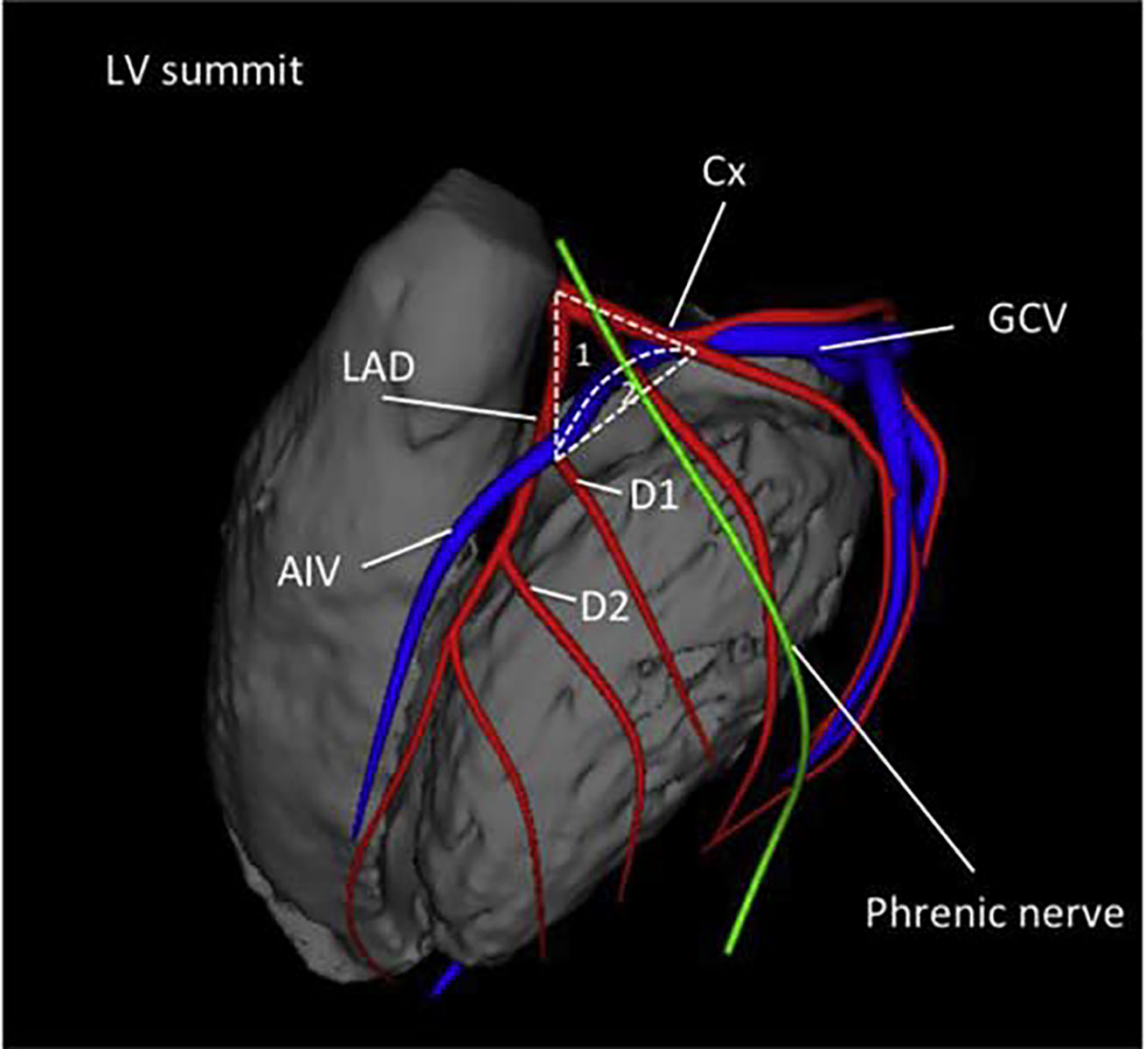
Anatomical boundaries of the LV summit, with the inaccessible [1] and accessible [2] parts. Shown are the left anterior descending artery (LAD), the circumflex artery (Cx), the great cardiac vein (GCV), the anterior interventricular vein (AIV), and the first and second diagonal branch of the LAD (D1, D2).

**Figure 8:**
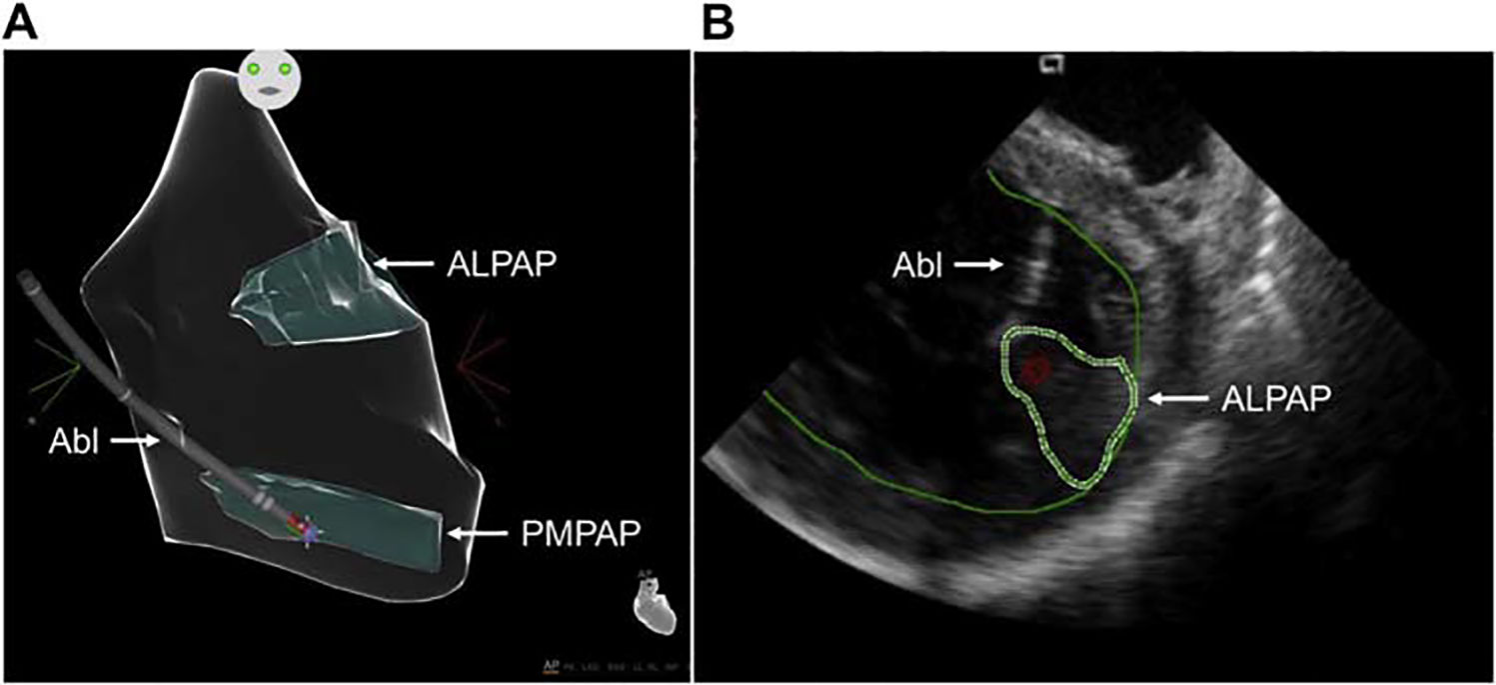
Intraprocedural imaging during ablation of papillary muscle arrhythmias. (A): Anatomical map of the left ventricle (CARTO, Biosense Webster) showing contact of the ablation catheter (Abl) with the posteromedial papillary muscle (PMPAP). (B): Intracardiac echocardiogram showing real-time visualization of the ablation catheter during ablation on the anterolateral papillary muscle (ALPAP).

**Figure 9:**
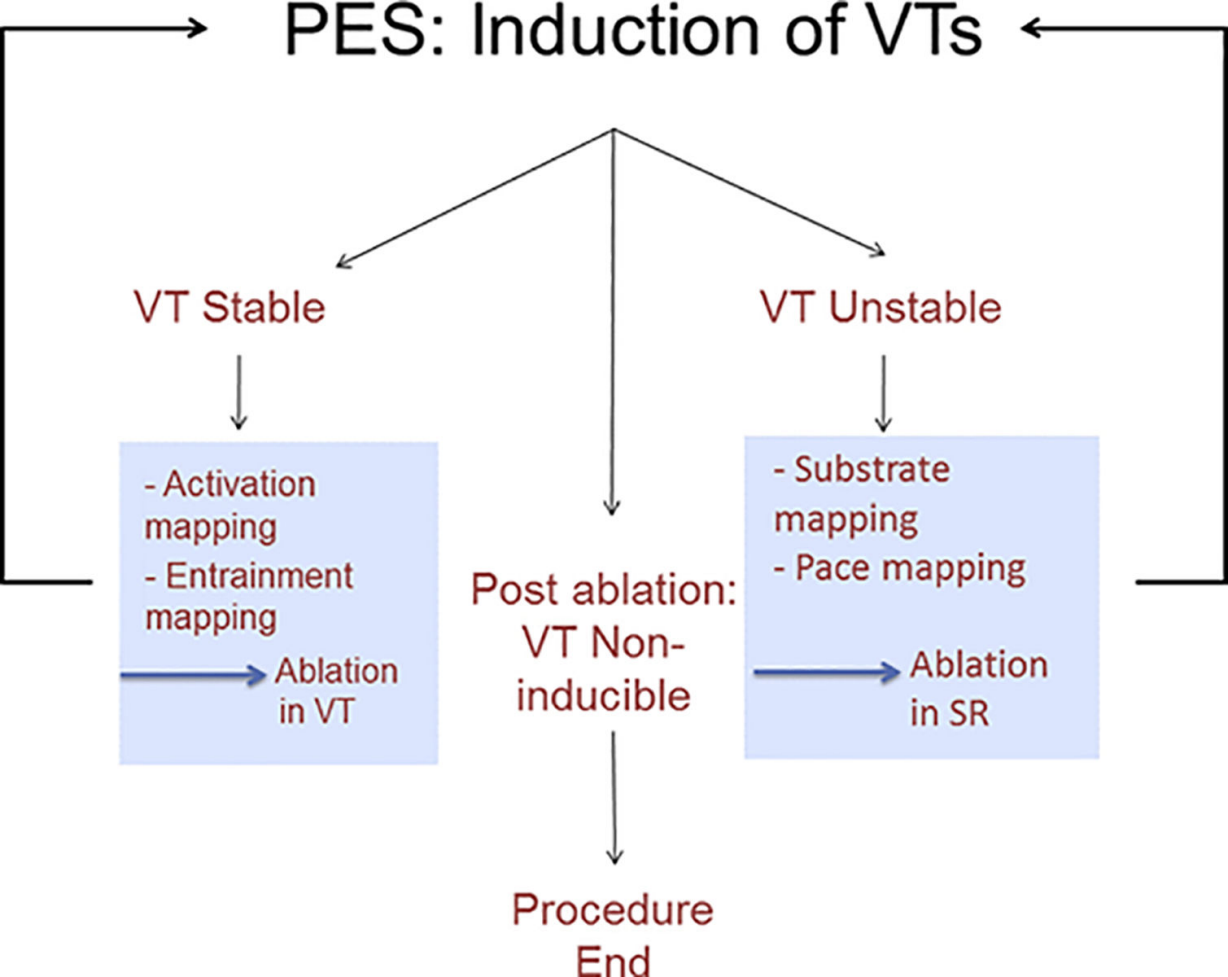
Overview of the workflow for catheter ablation of VT in patients with IHD. Not all of these steps might be required, and steps can be performed in a different sequence. For instance, repeat VT induction can be deferred in patients with hemodynamic instability. In addition, the operator might have to adapt to events that arise during the case, for instance, to take advantage of spontaneous initiation of stable VT during substrate mapping and switch to activation mapping. IHD = ischemic heart disease; PES = programmed electrical stimulation; SR = sinus rhythm; VT = ventricular tachycardia.

**Figure 10:**
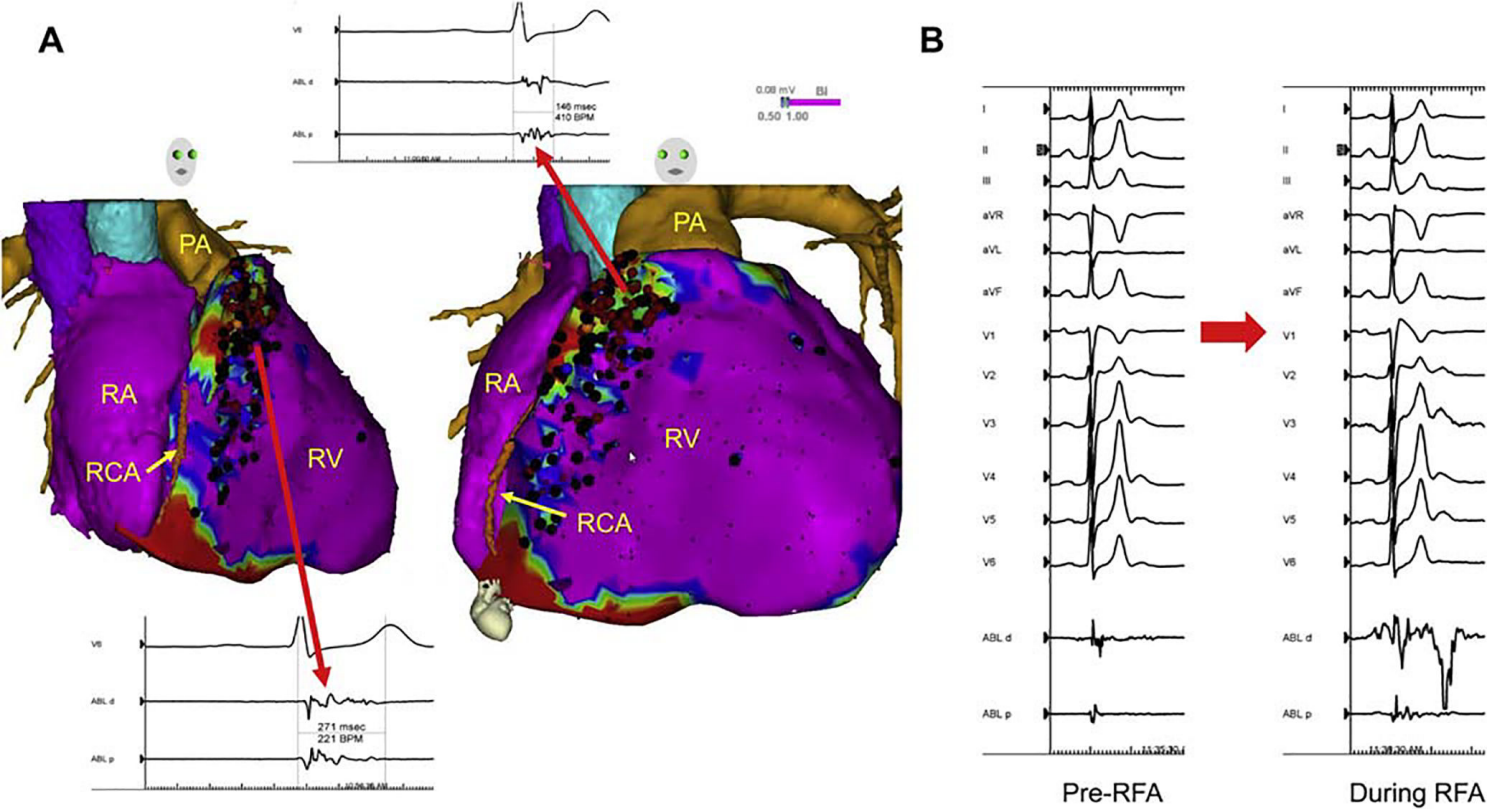
Epicardial substrate ablation in a patient with Brugada syndrome and appropriate ICD shocks for VF. Image integration of a preacquired CT with the electroanatomical epicardial substrate map is shown in (A). Purple represents bipolar voltage >1.5 mV. Fractionated potentials (arrows) are tagged with black dots, and a representative example is displayed. Widespread fractionated potentials were recorded from the epicardial aspect of the RVOT extending down into the basal RV body. Ablation lesions are tagged with red dots. Some fractionated potentials could not be ablated due to the proximity of the acute marginal branches of the right coronary artery. Panel (B) shows the significant transient accentuation of the Brugada ECG pattern during the application of radiofrequency energy at one of these sites. CT = computed tomography; ECG = electrocardiogram; ICD = implantable cardioverter defibrillator; PA = pulmonary artery; RA = right atrium; RCA = right coronary artery; RFA = radiofrequency ablation; RV = right ventricle; RVOT = right ventricular outflow tract; VF = ventricular fibrillation.

**Figure 11:**
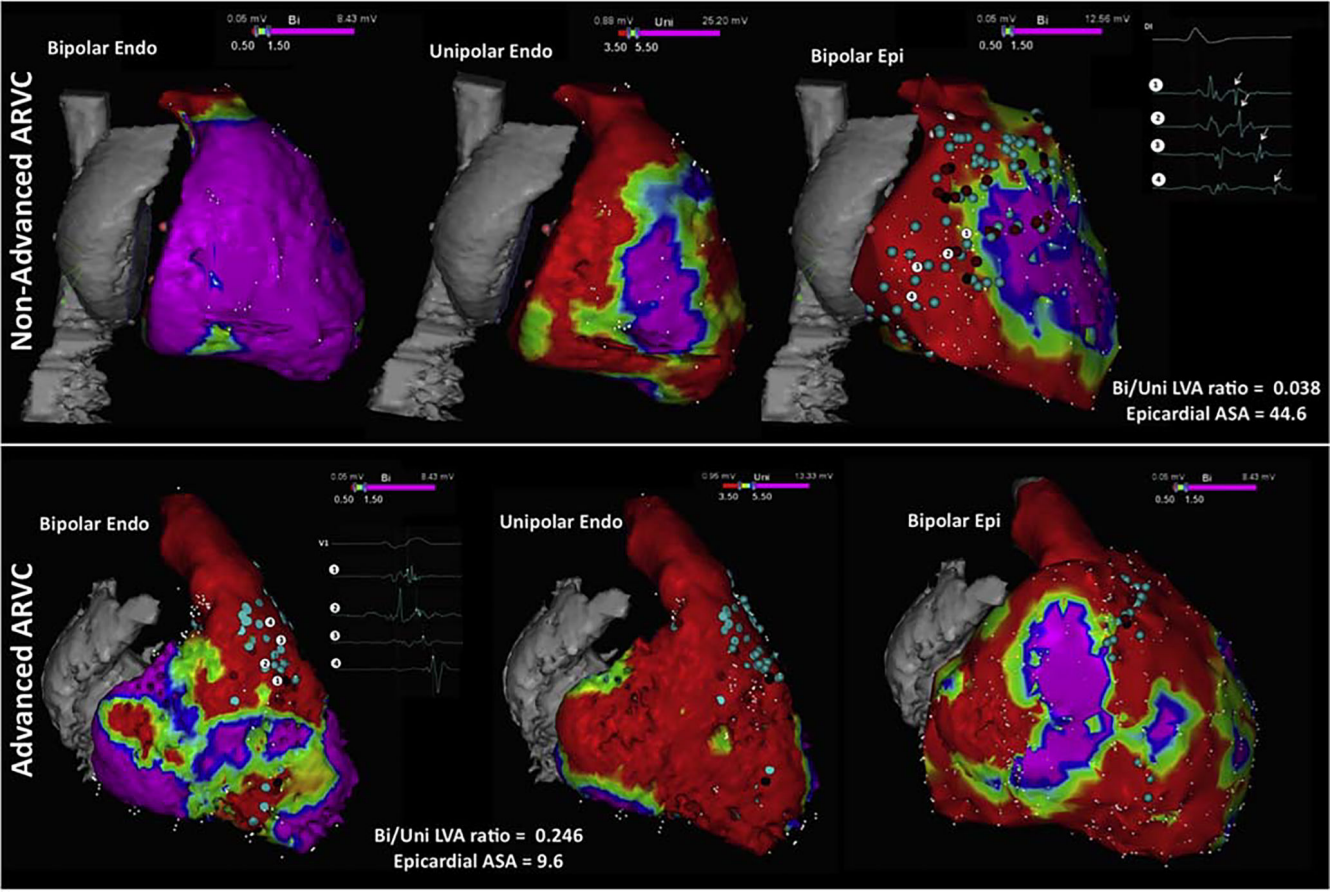
Right ventricular voltage maps from cases of moderate (upper row) and advanced (lower row) arrhythmogenic right ventricular cardiomyopathy (ARVC) are shown. Purple represents a voltage >1.5 mV in the bipolar maps (left and right) and >5.5 mV in the unipolar maps (center); red represents a voltage <0.5 mV in the bipolar maps and <3.5 mV in the unipolar maps. Moderate ARVC is defined as having a bipolar/unipolar low-voltage area ratio of <0.23 and is associated with epicardial arrhythmogenic substrate area (ASA) (defined by the presence of electrograms with delayed components of >10 cm^2^. Advanced ARVC displays a bipolar/unipolar endocardial low-voltage area of ≥0.23, which is associated with an epicardial arrhythmogenic substrate area of ≤10 cm^2^. Adapted with permission from Oxford University Press (Berruezo et al. Europace 2017;19:607–616).

**Figure 12:**
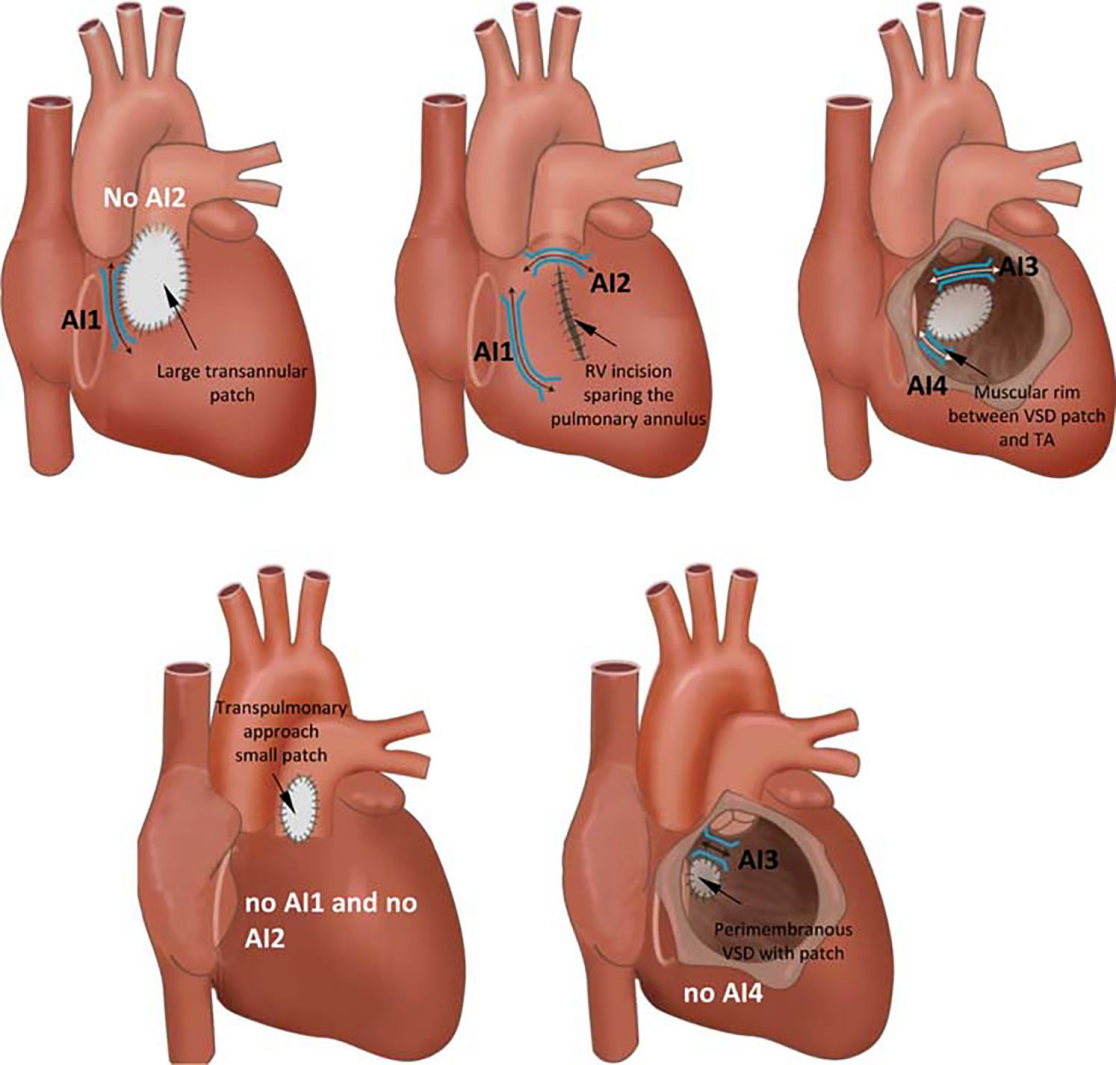
Anatomical isthmuses (AI) in repaired tetralogy of Fallot according to the surgical approach and variation of the malformation. RV = right ventricular; TA = tricuspid annulus; VSD = ventricular septal defect.

This section is designed as a “how-to” section that details the procedural steps of VT ablation in different patient populations ranging from ablation of PVCs in patients without heart disease to ablation of VT/VF in patients with different types of SHD (Figures 7–12 and Tables 5–8). Bullet points summarize the key points in this section.

### 9.1. Ablation Power Sources and Techniques

**Key Points**

- An impedance drop ≥10 ohms or a contact force ≥10 g is commonly used as a target for radiofrequency energy delivery.
- The use of half normal saline generates larger ablation lesions but can result in steam pops.
- Simultaneous bipolar or unipolar ablation can result in larger ablation lesions.
- Cryoablation can be beneficial for achieving more stable contact on the papillary muscles.
- Ethanol ablation can generate lesions in areas where the arrhythmogenic substrate cannot be otherwise reached, provided that suitable target vessels are present.
- Stereotactic radiotherapy is an emerging alternative to ablation, requiring identification of a region of interest that can be targeted prior to the radiation treatment.

### 9.2. Idiopathic Outflow Tract Ventricular Arrhythmia

**Key Points**

- The RVOT, pulmonary arteries, SVs, LV epicardium and endocardium contain most of the out_fl_ow tract arrhythmias.
- Activation mapping and pace mapping can be used to guide ablation in the RVOT.
- Imaging of coronary artery ostia is essential before ablation in the aortic SVs.
- The LV summit is a challenging site of origin, often requiring mapping and/or ablation from the RVOT, LVOT, SVs, coronary venous system, and sometimes the epicardial space.
- Deep intraseptal VA origins can be challenging to reach.

### 9.3. Idiopathic Nonoutflow Tract Ventricular Arrhythmia

**Key Points**

- VAs originating from the papillary muscles can be challenging due to multiple morphologies of the VA and the difficulty in achieving and maintaining sufficient contact during ablation.
- VAs originate in LV papillary muscles more often than in RV papillary muscles; they more often originate from the posteromedial than the anterolateral papillary muscle and occur more often at the tip than at the base.
- Pace mapping is less accurate than in other focal VAs.
- ICE is particularly useful for assessing contact and stability.
- Cryoablation can also aid in catheter stability during lesion delivery.

### 9.4. Bundle Branch Reentrant Ventricular Tachycardia and Fascicular Ventricular Tachycardia

**Key Points**

- Bundle branch reentry can occur in a variety of patients in whom the conduction system can be affected, including patients with dilated cardiomyopathy (DCM), valvular heart disease, myocardial infarction, myotonic dystrophy, Brugada syndrome, and ARVC, among others.
- Ablation of either the right or left bundle branch eliminates bundle branch reentrant ventricular tachycardia (BBRVT) but does not eliminate other arrhythmic substrates.
- A correct diagnosis of BBRVT is crucial and should employ established criteria prior to ablation of either of the bundle branches.
- Ablation of the AV node does not cure BBRVT.
- Ablation of either bundle branch does not cure interfascicular VT.
- For posterior fascicular VTs, the P1 potential is targeted during VT; if P1 cannot be identified or VT is not tolerated, an anatomical approach can be used.
- Purkinje fibers can extend to the papillary muscles, and these can be part of the VT circuit.
- For anterior fascicular VTs, the P1 potential is targeted with ablation.
- Focal nonreentrant fascicular VT is infrequent and can occur in patients with IHD; however, it cannot be induced with programmed stimulation, and the target is the earliest Purkinje potential during VT.

### 9.5. Postinfarction Ventricular Tachycardia

**Key Points**

- In cases of multiple inducible VTs, the clinical VT should be preferentially targeted.
- Elimination of all inducible VTs reduces VT recurrence and is associated with prolonged arrhythmia-free survival.
- For tolerated VTs, entrainment mapping allows for focal ablation of the critical isthmus.
- For nontolerated VTs, various ablation strategies have been described, including targeting abnormal potentials, matching pace mapping sites, areas of slow conduction, linear lesions, and scar homogenization.
- Imaging can be beneficial in identifying the arrhythmogenic substrate.
- Epicardial ablation is infrequently required, but epicardial substrate is an important reason for VT recurrence after VT ablation in patients with prior infarcts.

### 9.6. Dilated Cardiomyopathy

**Key Points**

- Identifying the location and extent of scarring on CMR is beneficial in procedural planning and has improved the outcomes of ablation in patients with DCM.
- The ablation strategy is similar to postinfarction VT.
- An intramural substrate is more frequently encountered in DCM than in postinfarction patients and requires a different ablation strategy than for patients with either epicardial or endocardial scarring.
- Epicardial ablation is beneficial if the scar is located in the epicardium of the LV free wall.
- For intramural circuits involving the septum, epicardial ablation is not beneficial.
- In the absence of CMR, unipolar voltage mapping has been described as a method to indicate a deeper-seated scar.

### 9.7. Ventricular Tachycardia Ablation in Hypertrophic Cardiomyopathy

**Key Points**

- Polymorphic VT and VF are the most common VAs in HCM; monomorphic VT is less common.
- The arrhythmogenic substrate in HCM often involves the septum but can extend to the epicardium, often necessitating combined endocardial and epicardial ablation procedures to eliminate the VT.
- VT associated with apical aneurysms is often ablated endocardially.

### 9.8. Brugada Syndrome

**Key Points**

- PVC-triggered VF or polymorphic VT are the most prevalent VAs that motivate device therapy in patients with Brugada syndrome.
- Monomorphic VT is less frequent but can be caused by BBRVT in patients with Brugada syndrome.
- The arrhythmogenic substrate is located in the RV epicardium and can be demonstrated by sodium channel blockers.
- Ablation targets include fractionated prolonged electrograms on the epicardial aspect of the RV.

### 9.9. Polymorphic Ventricular Tachycardia/Ventricular Fibrillation Triggers

**Key Points**

- Recurrent PVC-induced VF is most often triggered by PVCs originating from Purkinje fibers, located in the RVOT, the moderator band, or the LV.
- Patients with a single triggering PVC are better ablation candidates; however, there are often multiple triggers.
- Patients with healed myocardial infarction often require extensive ablation of the Purkinje fiber system within or at the scar border.
- Ischemia should be ruled out as a trigger for VF prior to ablation.

### 9.10. Arrhythmogenic Right Ventricular Cardiomyopathy

**Key Points**

- The arrhythmogenic substrate in ARVC is located in the epicardium and can involve the endocardium in advanced stages.
- The most commonly affected areas are the subtricuspid and RV outflow regions.
- LV involvement is not uncommon.
- Endocardial-epicardial ablation is often required and results in higher acute success and lower recurrence rates compared with endocardial ablation alone.
- Conventional mapping and ablation techniques, including entrainment mapping of tolerated VT, pace mapping, and substrate ablation, are used.

### 9.11. Mapping and Ablation in Congenital Heart Disease

**Key Points**

- Patients with a VT substrate after congenital heart defect surgery include those with repaired tetralogy of Fallot, repaired ventricular septal defect, and repaired d-transposition of the great arteries (D-TGA), as well as Ebstein’s anomaly among other disease processes.
- VT isthmuses are often located between anatomical barriers and surgical incisions or patch material.
- An anatomical isthmus can be identified and targeted during sinus rhythm.
- For tolerated VTs, entrainment mapping is the method of choice for identifying critical components of the reentry circuit.

### 9.12. Sarcoidosis

**Key Points**

- The arrhythmogenic substrate in cardiac sarcoidosis is often intramurally located but can include the endocardium and epicardium.
- A CMR is beneficial in planning an ablation procedure in cardiac sarcoidosis.
- The arrhythmogenic substrate can be complex and can include areas of active inflammation and chronic scarring.
- The VT recurrence rate after ablation is high.

### 9.13. Chagas Disease

**Key Points**

- The pathogenesis of Chagas disease is poorly understood but often results in an inferolateral LV aneurysm.
- The arrhythmogenic substrate is located intramurally and on the epicardial surface, often necessitating an epicardial ablation procedure.

### 9.14. Miscellaneous Diseases and Clinical Scenarios With Ventricular Tachycardia

**Key Points**

- Lamin cardiomyopathy often has a poor prognosis, progressing to end-stage heart failure.
- VT ablation is challenging due to intramural substrates.
- VT recurrence rate is high after ablations.
- VT in patients with noncompaction tends to originate from regions of noncompacted myocardium where scar can be identified in the midapical LV.
- VT ablation in patients with LV assist device can be challenging due to the limitation of preprocedural imaging, and the electromagnetic noise generated by the LV assist device.

### 9.15. Surgical Therapy

**Key Points**

- Surgery-facilitated access to the epicardium via a limited subxiphoid incision can be helpful in the case of adhesions.
- Cryoablation via thoracotomy is possible for posterolateral substrates and via sternotomy for anterior substrates.

### 9.16. Sympathetic Modulation

**Key Points**

- Sympathetic modulation targeting the stellate ganglia by video-assisted thoracoscopy may be considered for failed VT ablation procedures or VF storms.
- A temporary effect can be obtained with the percutaneous injection or infusion of local anesthetics.

### 9.17. Endpoints of Catheter Ablation of Ventricular Tachycardia

**Key Points**

- Noninducibility of VT by PES after ablation is a reasonable endpoint and predictor for VT recurrence after VT ablation in patients with SHD.
- Due to the limitations of programmed stimulation, endpoints other than noninducibility have been described, including elimination of excitability, elimination of late potentials or local abnormal ventricular activity, dechanneling, substrate homogenization, core isolation, image-guided ablation, and anatomically fixed substrate ablation.

## 10 Postprocedural Care

**Figure 13:**
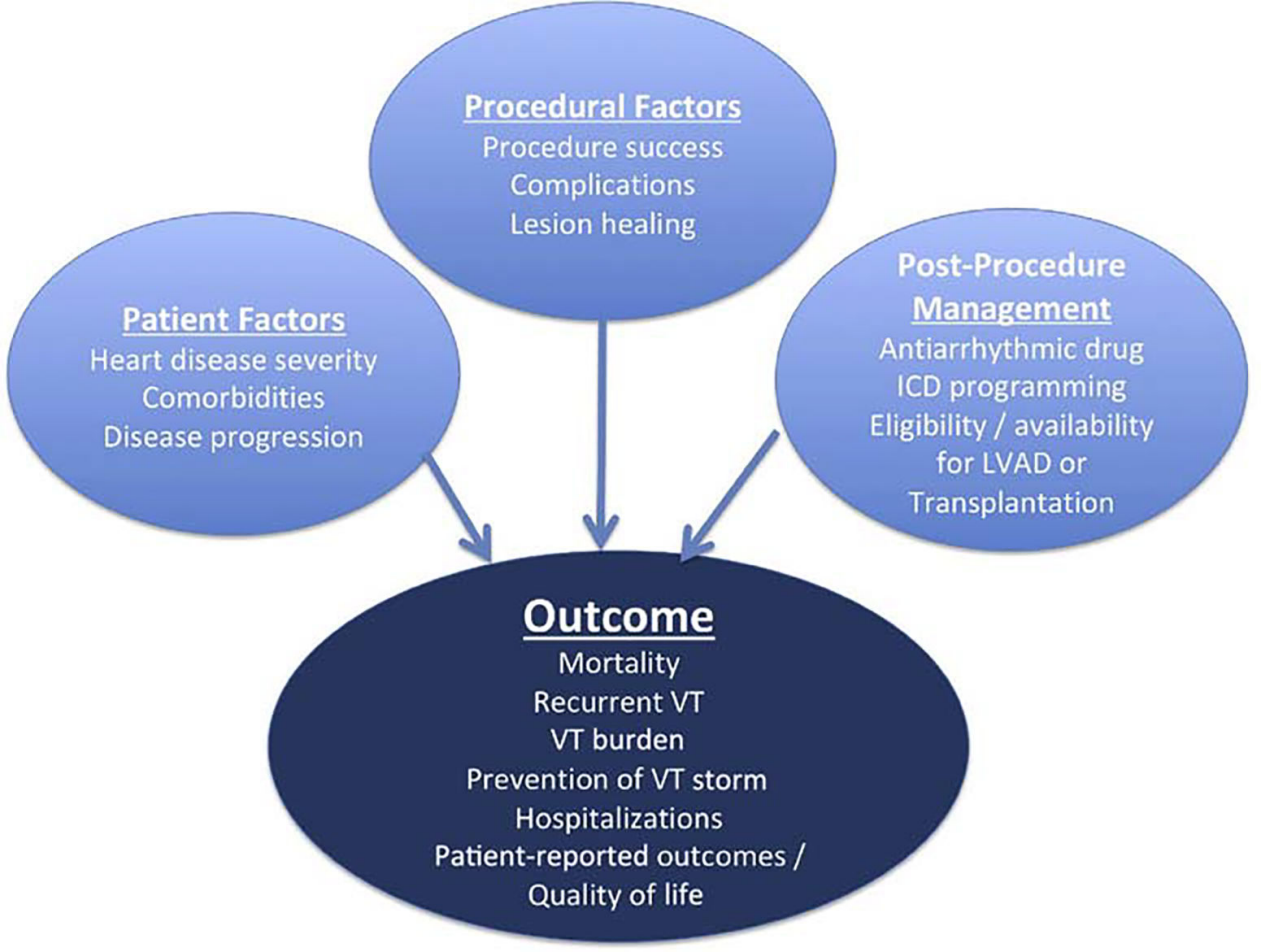
Factors influencing outcomes post VA ablation. ICD = implantable cardioverter defibrillator; LVAD = left ventricular assist device; VA = ventricular arrhythmia; VT = ventricular tachycardia.

Access-related issues, anticoagulation (Table 9), and complications (Table 10), as well as the management thereof, are reviewed in this section. Furthermore, assessment of outcomes and determinants of outcomes are detailed (Figure 13).

### 10.1. Postprocedural Care: Access, Anticoagulation, Disposition

#### 10.1.1. Postprocedural Care: Access

**Table:** Recommendations for management of venous access sites after catheter ablation of VA

COR	LOE	Recommendations	References

**I**	**A**	**1. Manual compression is effective in achieving hemostasis after venous access for VT ablation.**	S10.1.1.1–S10.1.1.3
**IIa**	**B-R**	**2. Venous access closure using temporary purse-string or figure-of-8 suture techniques can be useful in achieving faster hemostasis and earlier ambulation and reducing pain or discomfort associated with hemostasis compared to manual compression.**	S10.1.1.1–S10.1.1.2

**Table:** Recommendation for management of arterial access sites after catheter ablation of VA

COR	LOE	Recommendation	References

**I**	**A**	**1. Achieving arterial access site hemostasis using either manual compression or a vascular closure device is recommended.**	S10.1.1.4,S10.1.1.5

**Table:** Recommendations for management of epicardial access sites after catheter ablation of VA

COR	LOE	Recommendations	References

**I**	**C-EO**	**1. If pericardial bleeding or cardiac tamponade has occurred during epicardial VT ablation, a pericardial drain should be left in place until bleeding has resolved.**	
**IIa**	**B-NR**	**2. The instillation of intrapericardial corticosteroids can be effective in reducing pericarditic chest pain after epicardial VT mapping or ablation.**	S10.1.1.6,S10.1.1.7
**IIa**	**B-NR**	**3. To reduce pericardial pain after epicardial VT ablation, unless pericardial bleeding or cardiac tamponade has occurred, it is reasonable to remove all pericardial access sheaths at the end of the procedure.**	S10.1.1.6,S10.1.1.7
**IIb**	**C-EO**	**4. Leaving a pericardial drain in place might be reasonable in patients at high risk for late bleeding or cardiac tamponade after epicardial VT ablation.**	

#### 10.1.2. Postprocedural Care: Anticoagulation

**Table:** Recommendations for anticoagulation after VA ablation procedures

COR	LOE	Recommendations	References

**IIa**	**C-LD**	**1. After less extensive endocardial VT ablation, treatment with an antiplatelet agent for a limited period of time is reasonable.**	S10.1.2.1,S10.1.2.2
**IIa**	**C-LD**	**2. Heparin reversal with protamine for sheath removal after ablation is reasonable.**	S10.1.2.3,S10.1.2.4
**IIb**	**C-LD**	**3. After extensive endocardial VT ablation, treatment with an oral anticoagulant for a limited period of time might be reasonable.**	S10.1.2.1,S10.1.2.2
**IIb**	**C-EO**	**4. The use of heparin bridging after endocardial VT ablation may be considered but can be associated with an increased risk of periprocedural bleeding.**	

### 10.2. Incidence and Management of Complications

### 10.3. Hemodynamic Deterioration and Proarrhythmia

**Table:** Recommendation for echocardiography after VA ablation

COR	LOE	Recommendation	Reference

**I**	**C-LD**	**1. Echocardiography should be performed in case of hemodynamic deterioration post-VT ablation to assess for pericardial effusion and cardiac tamponade.**	S10.3.1

### 10.4. Follow-up of Patients Post Catheter Ablation of Ventricular Tachycardia

**Table:** Recommendation for noninvasive programmed stimulation after catheter ablation of VT

COR	LOE	Recommendation	References

**IIa**	**B-NR**	**1. Noninvasive programmed stimulation can be useful in the several days following VT catheter ablation to inform further management, including ICD programming, predicting the risk of VT recurrence, and/or considering a repeat VT catheter ablation.**	S10.4.1,S10.4.2

## 11 Training and Institutional Requirements and Competencies

This section contains the general training and institutional requirements with an emphasis on lifelong learning, professionalism, and acquisition and maintenance of knowledge and skills. In addition, institutional requirements for specific procedures are reviewed.

### 11.1. Training Requirements and Competencies for Catheter Ablation of Ventricular Arrhythmias

**Table:** Recommendation for training requirements and competencies for catheter ablation of VA

COR	LOE	Recommendation

**I**	**C-EO**	**1. For clinical cardiac electrophysiologists who perform catheter ablation for VAs, appropriate advanced training and continued lifelong learning is recommended.**

### 11.2. Institutional Requirements for Catheter Ablation of Ventricular Tachycardia

**Table:** Recommendations for institutional requirements for catheter ablation of VT

COR	LOE	Recommendations

**I**	**C-EO**	**1. Patients with certain underlying medical conditions and comorbidities undergoing complex VA ablations who are deemed to have increased procedural risk should undergo procedures in a hospital-based electrophysiology laboratory.**
**I**	**C-EO**	**2. Onsite interventional cardiology expertise is recommended for electrophysiology procedures requiring coronary imaging to delineate coronary anatomy for epicardial ablation, aortography to delineate coronary ostia for SV VT ablation, and need for placement of HS devices.**
**I**	**C-EO**	**3**. **Onsite cardiothoracic surgical backup is recommended for electrophysiology procedures requiring pericardial access due to the potential need for emergent sternotomy and cardiopulmonary bypass.**
**I**	**C-EO**	**4. Availability of anesthesia personnel is recommended for all patients undergoing catheter ablation of VAs.**

## 12 Future Directions

This section summarizes ongoing trials and the need for prospective evaluation of different clinical problems. It further reviews recent advances and limitations of various mapping techniques and addresses unanswered questions requiring future investigations.

## Supplementary Material

supplementary material Appen

## Figures and Tables

**Table 1:** ACC/AHA Recommendation System: Applying Class of Recommendation and Level of Evidence to Clinical Strategies, Interventions, Treatments, and Diagnostic Testing in Patient Care*

**CLASS (STRENGTH) OF RECOMMENDATION**	

**CLASS I (STRONG)**	**Benefit >>> Risk**

Suggested phrases for writing recommendations:	
■ Is recommended	
■ Is indicated/useful/effective/beneficial	
■ Should be performed/administered/other	
■ Comparative-Effectiveness Phrases†:	
○ Treatment/strategy A is recommended/indicated in preference to treatment B	
○ Treatment A should be chosen over treatment B	

**CLASS IIa (MODERATE)**	**Benefit >> Risk**

Suggested phrases for writing recommendations:	
■ Is reasonable	
■ Can be useful/effective/beneficial	
■ Comparative-Effectiveness Phrases†:	
○ Treatment/strategy A is probably recommended/indicated in preference to treatment B	
○ It is reasonable to choose treatment A over treatment B	

**CLASS IIb (WEAK)**	**Benefit ≥ Risk**

Suggested phrases for writing recommendations:	
■ May/might be reasonable	
■ May/might be considered	
■ Usefulness/effectiveness is unknown/unclear/uncertain or not well established	

**CLASS III: No Benefit (MODERATE) *(Generally, LOE A or B use only)***	**Benefit = Risk**

Suggested phrases for writing recommendations:	
■ Is not recommended	
■ Is not indicated/useful/effective/beneficial	
■ Should not be performed/administered/other	

**CLASS III: Harm (STRONG)**	**Risk > Benefit**

Suggested phrases for writing recommendations:	
■ Potentially harmful	
■ Causes harm	
■ Associated with excess morbidity/mortality	
■ Should not be performed/administered/other	

**LEVEL (QUALITY) OF EVIDENCE** ‡	

**LEVEL A**	

■ High-quality evidence‡ from more than 1 RCTs	
■ Meta-analyses of high-quality RCTs	
■ One or more RCTs corroborated by high-quality registry studies	

**LEVEL B-R**	**(Randomized)**

■ Moderate-quality evidence‡ from 1 or more RCTs	
■ Meta-analyses of moderate-quality RCTs	

**LEVEL B-NR**	**(Nonrandomized)**

■ Moderate-quality evidence‡ from 1 or more well-designed, well-executed nonrandomized studies, observational studies, or registry studies	
■ Meta-analyses of such studies	

**LEVEL C-LD**	**(Limited Data)**

■ Randomized or nonrandomized observational or registry studies with limitations of design or execution	
■ Meta-analyses of such studies	
■ Physiological or mechanistic studies in human subjects	

**LEVEL C-EO**	**(Expert Opinion)**

Consensus of expert opinion based on clinical experience	

COR and LOE are determined independently (any COR may be paired with any LOE).

A recommendation with LOE C does not imply that the recommendation is weak. Many important clinical questions addressed in guidelines do not lend themselves to clinical trials. Although RCTs are unavailable, there may be a very clear clinical consensus that a particular test or therapy is useful or effective.

* The outcome or result of the intervention should be specifed (an improved clinical outcome or increased diagnostic accuracy or incremental prognostic informaron).

† For comparative-effectiveness recommendations (COR I and IIa; LOE A and B only), studies that support the use of comparator verbs should involve direct comparisons of the treatments or strategies being evaluated.

‡ The method of assessing quality is evolving, including the application of standardized, widely used, and preferably validated evidence grading tools; and for systematic reviews, the incorporaron of an Evidence Review Committee.

COR indicates Class of Recommendation; EO, expert opinion; LD, limited data; LOE, Level of Evidence; NR, nonrandomized; R, randomized; and RCT, randomized controlled trial.

Reproduced with permission of the American College of Cardiology (ACC) and the American Heart Association (AHA) (S1.2.2).

**Table 2:** Definitions

**Clinical Characteristics**
***Clinical ventricular tachycardia (VT)*:** VT that has occurred spontaneously based on analysis of 12-lead electrocardiogram (ECG) QRS morphology.
***Hemodynamically unstable VT*:** causes hemodynamic compromise requiring prompt termination.
***Idiopathic VT*:** used to indicate VT that is known to occur in the absence of clinically apparent structural heart disease (SHD).
***Idioventricular rhythm*:** three or more consecutive beats at a rate of up to 100 per minute that originate from the ventricles independent of atrial or atrioventricular (AV) nodal conduction. Although various arbitrary rates have been used to distinguish it from VT, the mechanism of ventricular rhythm is more important than the rate. Idioventricular rhythm can be qualified as “accelerated” when the rate exceeds 40 bpm.
***Incessant VT*:** continuous sustained VT that recurs promptly despite repeated intervention for termination over several hours.
***Nonclinical VT*:** VT induced by programmed electrical stimulation (PES) that has not been documented previously.
***Nonsustained VT*:** terminates spontaneously within 30 seconds.
***PVC*:** premature ventricular complex; it is an early ventricular depolarization with or without mechanical contraction. We recommend avoiding the use of the terms “ventricular premature depolarization” and “premature ventricular contraction” to standardize the literature and acknowledge that early electrical activity does not necessarily lead to mechanical contraction.
***Presumptive clinical VT*:** similar to a spontaneous VT based on rate, limited ECG, or electrogram data available from ICD interrogation, but without the 12-lead ECG documentation of spontaneous VT.
***PVC burden*:** the amount of ventricular extrasystoles, preferably reported as the % of beats of ventricular origin of the total amount of beats over a 24-hour recording period.
***Repetitive monomorphic VT*:** continuously repeating episodes of self-terminating nonsustained VT.
***Sustained VT*:** continuous VT for 30 seconds, or which requires an intervention for termination (such as cardioversion).
***VT*:** a tachycardia (rate >100 bpm) with 3 or more consecutive beats that originates from the ventricles independent of atrial or AV nodal conduction.
***VT storm*:** three or more separate episodes of sustained VT within 24 hours, each requiring termination by an intervention.
**VT Morphologies**
***Monomorphic VT*:** a similar QRS configuration from beat to beat (Figure 1A). Some variability in QRS morphology at initiation is not uncommon, followed by stabilization of the QRS morphology.
***Monomorphic VT with indeterminate QRS morphology*:** preferred over ***ventricular flutter;*** it is a term that has been applied to rapid VT that has a sinusoidal QRS configuration that prevents identification of the QRS morphology.
***Multiple monomorphic VTs*:** more than one morphologically distinct monomorphic VT, occurring as different episodes or induced at different times.
***Pleomorphic VT*:** has more than one morphologically distinct QRS complex occurring during the same episode of VT, but the QRS is not continuously changing (Figure 1B).
***Polymorphic VT*:** has a continuously changing QRS configuration from beat to beat, indicating a changing ventricular activation sequence (Figure 1C).
***Right bundle branch block (RBBB)- and left bundle branch block (LBBB)-like VT configurations*:** terms used to describe the dominant deflection in V1, with a dominant R wave described as “RBBB-like” and a dominant S wave with a negative final component in V1 described as “LBBB-like” configurations.
***Torsades de pointes*:** a form of polymorphic VT with continually varying QRS complexes that appear to spiral around the baseline of the ECG lead in a sinusoidal pattern. It is associated with QT prolongation.
***Unmappable VT*:** does not allow interrogation of multiple sites to define the activation sequence or perform entrainment mapping; this could be due to hemodynamic intolerance that necessitates immediate VT termination, spontaneous or pacing-induced transition to other morphologies of VT, or repeated termination during mapping.
***Ventricular fibrillation (VF)*:** a chaotic rhythm defined on the surface ECG by undulations that are irregular in both timing and morphology, without discrete QRS complexes.
**PVC Morphologies**
***Monomorphic PVC*:** PVCs felt reasonably to arise from the same focus. Slight changes in QRS morphology due to different exit sites from the same focus can be present.
***Multiple morphologies of PVC*:** PVCs originating from several different focal locations.
***Predominant PVC morphology*:** the one or more monomorphic PVC morphologies occurring most frequently and serving as the target for ablation.
**Mechanisms**
***Focal VT*:** a point source of earliest ventricular activation with a spread of activation away in all directions from that site. The mechanism can be automaticity, triggered activity, or microreentry.
***Scar-related reentry*:** arrhythmias that have characteristics of reentry that originate from an area of myocardial scar identified from electrogram characteristics or myocardial imaging. Large reentry circuits that can be defined over several centimeters are commonly referred to as “macroreentry.”

AV = atrioventricular; ECG = electrocardiogram; ICD = implantable cardioverter defibrillator; LBBB = left bundle branch block; PES = programmed electrical stimulation; PVC = premature ventricular complex; RBBB = right bundle branch block; SHD = structural heart disease; VT = ventricular tachycardia.

**Table 3:** Anatomical terminology

Term	Definition

RV inflow	The part of the right ventricle (RV) containing the tricuspid valve, chordae, and proximal RV.
RV outflow tract (RVOT)	The conus or infundibulum of the RV, derived from the bulbus cordis. It is bounded by the supraventricular crest and the pulmonic valve.
Tricuspid annulus	Area immediately adjacent to the tricuspid valve, including septal, free wall, and para-Hisian regions.
Moderator band	A muscular band in the RV, typically located in the mid to apical RV, connecting the interventricular septum to the RV free wall, supporting the anterior papillary muscle. It typically contains a subdivision of the right bundle branch (RBB).
RV papillary muscles	Three muscles connecting the RV myocardium to the tricuspid valve via the tricuspid chordae tendineae, usually designated as septal, posterior, and anterior papillary muscles. The septal papillary muscle is closely associated with parts of the RBB.
Supraventricular crest	Muscular ridge in the RV between the tricuspid and pulmonic valves, representing the boundary between the conus arteriosus and the rest of the RV. The exact components and terminology are controversial; however, some characterize it as being composed of a parietal band that extends from the anterior RV free wall to meet the septal band, which extends from the septal papillary muscle to meet it.
Pulmonary valves	The pulmonic valve includes three cusps and associated sinus, variously named right, left, and anterior; or anterolateral right, anterolateral left, and posterior sinuses. The posterior-right anterolateral commissure adjoins the aorta (junction of the right and left aortic sinuses). Muscle is present in each of the sinuses, and VA can originate from muscle fibers located within or extending beyond the pulmonary valve apparatus.
Sinuses of Valsalva (SV), aortic cusps, aortic commissures	The right (R), left (L), and noncoronary aortic valve cusps are attached to the respective SV. The left sinus of Valsalva (LSV) is posterior and leftward on the aortic root. The noncoronary sinus of Valsalva (NCSV) is typically the most inferior and posterior SV, located posterior and rightward, superior to the His bundle, and anterior and superior to the paraseptal region of the atria near the superior AV junctions, typically adjacent to atrial myocardium. The right sinus of Valsalva (RSV) is the most anterior cusp and may be posterior to the RVOT infundibulum. VAs can also arise from muscle fibers at the commissures (connections) of the cusps, or from myocardium accessible to mapping and ablation from this location, especially from the RSV/LSV junction.
LV outflow tract (LVOT)	The aortic vestibule, composed of an infra-valvular part, bounded by the anterior mitral valve leaflet, but otherwise not clearly distinguishable from the rest of the LV; the aortic valve; and a supra-valvular part.
LV ostium	The opening at the base of the LV to which the mitral and aortic valves attach.
Aortomitral continuity (AMC); aortomitral curtain, or mitral-aortic intervalvular fibrosa	Continuation of the anteromedial aspect of the mitral annulus to the aortic valve; a curtain of fibrous tissue extending from the anterior mitral valve leaflet to the left and noncoronary aortic cusps. The AMC is connected by the left and right fibrous trigones to ventricular myocardium, the right fibrous trigone to the membranous ventricular septum.
Mitral valve annulus	Area immediately adjacent to the mitral valve. This can be approached endocardially, or epicardially, either through the coronary venous system or percutaneously.
LV papillary muscles	Muscles connecting the mitral valve chordae tendineae to the LV, typically with posteromedial and anterolateral papillary muscles. Papillary muscle anatomy is variable and can have single or multiple heads.
LV false tendon (or LV moderator band)	A fibrous or fibromuscular chord-like band that crosses the LV cavity, attaching to the septum, papillary muscles, trabeculations, or free wall of the LV. They may contain conduction tissue and may impede catheter manipulation in the LV.
Posterior-superior process	The posterior-superior process of the left ventricle (LV) is the most inferior and posterior aspect of the basal LV, posterior to the plane of the tricuspid valve. VAs originating from the posterior-superior process of the LV can be accessed from the right atrium, the LV endocardium, and the coronary venous system.
Endocardium	Inner lining of the heart.
Purkinje network	The specialized conduction system of the ventricles, which includes the His bundle, RBB and left bundle branches (LBB), and the ramifications of these, found in the subendocardium. The Purkinje system can generate focal or reentrant VTs, typically manifesting Purkinje potentials preceding QRS onset.
Interventricular septum	Muscular wall between the LV and RV.
Membranous ventricular septum	The ventricular septum beneath the RSV and NCSV, through which the penetrating His bundle reaches the ventricular myocardium.
LV summit	Triangular region of the most superior part of the LV epicardial surface bounded by the left circumflex coronary artery, the left anterior descending artery, and an approximate line from the first septal coronary artery laterally to the left AV groove. The great cardiac vein (GCV) bisects the triangle. An area superior to the GCV is considered to be inaccessible to catheter ablation due to proximity of the coronary arteries and overlying epicardial fat.
Crux of the heart (crux cordis)	Epicardial area formed by the junction of the AV groove and posterior interventricular groove, at the base of the heart, approximately at the junction of the middle cardiac vein and coronary sinus (CS) and near the origin of the posterior descending coronary artery.
Epicardium	The outer layer of the heart—the visceral layer of the serous pericardium.
Epicardial fat	Adipose tissue variably present over the epicardial surface around coronary arteries, LV apex, RV free wall, left atrial appendage, right atrial appendage, and AV and interventricular grooves.
Pericardial space or cavity	The potential space between the parietal and visceral layers of serous pericardium, which normally contains a small amount of serous fluid. This space can be accessed for epicardial procedures.
Parietal pericardium	The layer of the serous pericardium that is attached to the inner surface of the fibrous pericardium and is normally apposed to the visceral pericardium, separated by a thin layer of pericardial fluid.
Fibrous pericardium	Thick membrane that forms the outer layer of the pericardium.
Subxiphoid area	Area inferior to the xiphoid process; typical site for percutaneous epicardial access.
Phrenic nerve	The right phrenic nerve lays along the right atrium and does not usually pass over ventricular tissue. The course of the left phrenic nerve on the fibrous pericardium can be quite variable and may run along the lateral margin of the LV near the left obtuse marginal artery and vein; inferior, at the base of the heart; or anterior over the sternocostal surface over the L main stem coronary artery or left anterior descending artery.
Coronary sinus (CS) and branches	The CS and its branches comprise the coronary venous system with the ostium of the CS opening into the right atrium. Tributaries of the CS, which runs along the left AV groove, may be used for mapping. These include the anterior interventricular vein (AIV), which arises at the apex and runs along the anterior interventricular septum, connecting to the GCV that continues in the AV groove to the CS; the communicating vein located between aortic and pulmonary annulus; various posterior and lateral marginal branches or perforator veins; and the middle cardiac vein that typically runs along the posterior interventricular septum from the apex to join the CS or empty separately into the right atrium. The junction of the GCV and the CS is at the vein or ligament of Marshall (or persistent left superior vena cava, when present), and the valve of Vieussens (where present).

Anatomical terminology (S2.1–S2.9). See also Figures 3, 4, 7, and 8. AIV = anterior interventricular vein; AMC = aortomitral continuity; AV = atrioventricular; CS = coronary sinus; GCV = great cardiac vein; LBB = left bundle branch; LSV = left sinus of Valsalva; LV = left ventricle; LVOT = left ventricular outflow tract; NCSV = noncoronary sinus of Valsalva; RBB = right bundle branch; RSV = right sinus of Valsalva; RV = right ventricle; RVOT = right ventricular outflow tract; SV = sinus of Valsalva; VA = ventricular arrhythmia; VT = ventricular tachycardia.

**Table 4:** The PAAINESD Score, developed to predict the risk of periprocedural hemodynamic decompensation

Variable	Points

**P**ulmonary disease (COPD)	5
**A**ge >60	3
General **a**nesthesia	4
**I**schemic cardiomyopathy	6
**N**YHA class III/IV	6
**E**F <25%	3
VT **s**torm	5
**D**iabetes mellitus	3

The PAAINESD Score, developed to predict the risk of periprocedural hemodynamic decompensation, has values that range from 0 to 35 points (or 0 to 31 [PAINESD] when the modifiable intraprocedural variable “general anesthesia” is excluded) (Santangeli et al. Circ Arrhythm Electrophysiol 2015;8:68–75). COPD = chronic obstructive pulmonary disease; EF = ejection fraction; NYHA = New York Heart Association; VT = ventricular tachycardia.

**Table 5:** Types of bundle branch reentrant tachycardia

	Type A	Type B (Interfascicular tachycardia)	Type C

**ECG morphology**	LBBB pattern	RBBB pattern	RBBB pattern
**Anterograde limb**	RBB	LAF or LPF	LBB
**Retrograde limb**	LBB	LPF or LAF	RBB

LAF = left anterior fascicle; LBB = left bundle branch; LBBB = left bundle branch block; LPF = left posterior fascicle; RBB = right bundle branch; RBBB = right bundle branch block.

**Table 6:** Fascicular ventricular tachycardias

**I. Verapamil-sensitive fascicular reentrant VT**
**1. Left posterior type**
i. Left posterior septal fascicular reentrant VT
ii. Left posterior papillary muscle fascicular reentrant VT
**2. Left anterior type**
i. Left anterior septal fascicular reentrant VT
ii. Left anterior papillary muscle fascicular reentrant VT
**3. Upper septal type**
**II. Nonreentrant fascicular VT**

VT = ventricular tachycardia.

**Table 7:** Select recent radiofrequency catheter ablation studies in patients post myocardial infarction with a focus on substrate-based ablation strategies

Study	N	EF (%)	Prior CABG (%)	Inclusion	Access mapping catheter	Mapping strategy	Ablation strategy	Procedural endpoint	RF time procedural duration complications	VT recurrence and burden (follow-up)

Jais et al. (2012) (S9.5.1) Two centers observational	70	35 ± 10	NR	1) Sustained VT resistant to AAD therapy and requiring external cardioversion or ICD therapies 2) SHD with ischemic or nonischemic dilated cardiomyopathy Exclusions: 1) VA attributable to an acute or reversible cause 2) Repetitive PVCs or nonsustained VT without sustained VT	Retrograde in 61 pts (87%) Transseptal in 32 pts (46%); epicardial access in 21 pts (31%) Dual access encouraged 3.5-mm external irrigated ablation catheter; multielectrode mapping catheter in 50% endocardial procedures and in all epicardial procedures	1) PES and activation mapping of induced stable VTs 2) Substrate mapping for LAVAs — sharp high-frequency electrograms often of low amplitude, occurring during or after the far-field ventricular electrogram, sometimes fractionated or multicomponent, poorly coupled to the rest of the myocardium	1) Ablation of LAVA in SR 2) Ablation of tolerated VTs guided by entrainment and activation mapping 3) Remapping (in stable patients) with further ablation if residual LAVA or persistent inducibility	1) Complete LAVA elimination — achieved in 47 of 67 pts with LAVA (70.1%) 2) Noninducibility — achieved in 70%, similar if LAVA eliminated or not	RF time 23 ± 11 min Procedure time 148 ± 73 min Complications 6 pts (8.6%): tamponade or bleeding managed conservatively (3), RV perforation requiring surgical repair (1); 3 pts died within 24 h due to low-flow state (2) plus arrhythmia recurrence (1), PEA (1)	Combined endpoint of VT recurrence or death occurred in 39 pts (55.7%); 45% of pts with LAVA elimination and 80% of those without VT recurrence in 32 (46%); 32% of pts with LAVA elimination and 75% of those without 7 cardiac deaths (10%) over 22 months of median follow-up
Di Biase et al. (2015) (S9.5.2) VISTA trial Multicenter RCT	118	Group 1 33 ± 14 Group 2 32 ± 10	34%	1) Post-MI 2) Recurrent stable AAD refractory VT (symptomatic or requiring ICD therapy) Exclusion: syncope, cardiac arrest, prior failed ablation, renal failure, end-stage heart failure	Endocardial Epicardial when clinical VTs were inducible after endocardial ablation + no CABG Group 1: 11.7% Group 2: 10.3% 3.5-mm tip	1) Substrate mapping (BV ≤1.5 mV) + Group 1 2) PES and activation mapping/pace mapping for clinical and stable nonclinical VT (unstable VT not targeted)	Group 1: Clinical VT ablation, linear lesion to transect VT isthmus Group 2: Extensive substrate ablation targeting any abnormal potential (=fractionated and/or LP)	Group 1: Noninducibility of clinical VT — achieved in 100% Group 2: 1) Elimination of abnormal potentials 2) No capture from within the scar (20 mA) 3) Noninducibility of clinical VT — achieved in 100%	Group 1: RF time 35 ± 27 min Procedural time 4.6 ± 1.6 h Group 2: RF time 68 ± 27 min (*P* < .001) Procedural time 24.2 ± 1.3 h (*P* = .13) Complications 5%	VT recurrence at 12 months Group 1: 48.3% Group 2: 15.5% *P* < .001 Mortality at 12 months Group 1: 15% Group 2: 8.6% *P* = .21
Tilz et al. (2014) (S9.5.3) Single center observational	12 12/117 pts with post-MI VT	32 ± 13	—	1) Presence of a circumscribed dense scar (BV <1.5 mV, area <100 cm^2^) 2) Recurrent unmappable VT 3) Post-MI Exclusion: patchy scar/multiple scars	Endocardial 3.5-mm tip	1) PES 2) Substrate mapping: area of BV <1.5 mV + double, fractionated or LP 3) PES after ablation	Circumferential linear lesion along BZ (BV <1.5 mV) to isolate substrate	1) Lack of abnormal EGMs within area 2) No capture within area — achieved in 50% 3) Max. 40 RF lesion Noninducibility of any VT (no predefined endpoint) — observed in 92%	RF time 53 ± 15 min Procedure time 195 ± 64 min No complication	VT recurrence 33% Median follow-up 497 days
Tzou et al. (2015) (S9.5.4) Two centers observational	44 Post-MI 32 44/566 pts with SHD	31 ± 13	—	1) SHD 2) AAD refractory VT 3) Intention to achieve core isolation	Endocardial Epicardial post-MI 6% 3.5-mm tip Selected patients: multi-electrode catheters for exit block evaluation	1) BV mapping 2) PES 3) Activation mapping 4) Substrate mapping Dense scar BV <0.5 mV; BZ BV 0.5–1.5 mV/voltage channels/fractionated/LP; pace-match, S-QRS >40 ms 5) PES after core isolation	1) Circumferential linear lesion to isolate core (=confluent area of BV <0.5 mV area and regions with BV <1 mV harbouring VT-related sites 2) Targeting fractionated and LP within core 3) Targeting VT-related sites outside core (2 and 3 in 59%)	1) No capture of the ventricle during pacing inside core 2) Dissociation of isolated potentials — core isolation achieved in 70% post-MI 3) Noninducibility — achieved in 84%	RF lesions 111 ± 91 Procedure time 326 ± 121 min Complications 2.2% No death	VT recurrence 14% Follow-up 17.5 ± 9 months
Silberbauer et al. (2014) (S9.5.5) One center observational	160	28 ± 9.5 inducible after RFCA 34 ± 9.2 endpoint reached	22.5%	1) Post-MI 2) AAD refractory VT 3) First VT ablation at the center	Endocardial Combined endoepicardial (20%) — Clinical findings — Prior ablation — Research protocol 3.5-mm tip/4-mm tip	1) Substrate mapping: BV <1.5 mV + LP (=continuous, fragmented bridging to components after QRS offset/inscribing after QRS, no voltage cutoff) + early potentials (EP = fragmented <1.5 mV) Pace-match 2) PES 3) Activation mapping 4) PES after substrate ablation	1) Ablation mappable VT 2) Ablation of all LP LP present at baseline Endocardium 100/160 pts Epicardium 19/32 pts	1) Abolition of all LP — achieved at endocardium in 79 pts (49%), at epicardium 12/32 pts (37%) 2) Noninducibility of any VT — achieved in 88%	RF time endocardial median ≈25 min epicardial ≈6 min Procedure time Median 210–270 min Complications 3.1% In-hospital mortality 2.5%	VT recurrence 32% after median 82 (16–192) days VT recurrence according to endpoint 1+2 achieved (16.4%) Endpoint 2 achieved (46%) No endpoint achieved (47.4%)
Wolf et al. (2018) (S9.5.6) One center observational	159	34 ± 11	25%	1) Post-MI 2) First VT ablation 3) Recurrent, AAD refractory episodes VT	Endocardial Combined endoepicardial 27% — Epicardial access was encouraged — Epicardial ablation 27/46 pts 3.5-mm tip (70 pts) Multielectrode catheters (89 pts)	1) PES 2) Activation mapping 3) Substrate mapping: BV mapping (<1.5 mV) + LAVA (=sharp high-frequency EGMs, possibly of low amplitude, distinct from the far-field EGM occurring anytime during or after the far-field EGM 4) PES	1) Ablation of mappable VT 2) Ablation of LAVA (until local no capture) LAVA present at baseline Endocardium 141/157 pts Epicardium 36/46 pts	1) Abolition of LAVA — achieved in 93/146 pts (64%) 2) Noninducibility — achieved in 94/110 tested pts	RF time 36 ± 20 min Procedure time 250 ± 78 min Complications 7.5% (4 surgical interventions) Procedure-related mortality 1.3%	VT-free survival 55% during 47 months (33–82) Outcome according to endpoints: LAVA abolished vs not abolished 63% vs 44% VT-free survival at 1 year 73%
Berruezo et al. (2015) (S9.5.7) One center observational	101 Post-MI 75	36 ± 13	—	1) Scar-related VT	Endocardial Combined endoepicardial (27/101 pts, among post-MI not provided) — Endo no substrate/suggestive epi — CE-MRI — VT ECG 3.5-mm tip	1) Substrate mapping: BV (<1.5 mV) + EGMs with delayed components: identification of entrance (shortest delay) of conducting channels 2) PES 3) Activation mapping + pace-match	1) Scar dechanneling targeting entrance 2) Short linear lesions (eg, between scar and mitral annulus) 3) Ablation of VT-related sites — performed in 45%	1) Scar dechanneling — Achieved in 85 pts (84.2%) — Noninducible after 1) 55 pts (54.5%) 2) Noninducibility —achieved in 78%	RF time 24 ± 10 min only scar dechanneling (31 ± 18 min + additional RFCA) Procedure time 227 ± 69 min Complications 6.9% No death	VT recurrence 27% after a median follow-up of 21 months (11–29) 1-year VT-free survival according to endpoint: scar dechanneling complete vs incomplete (≈82% vs ≈65%)
Porta-Sánchez et al. (2018) (S9.5.8) Multicenter observational	20	33 ± 11	—	1) Post-MI 2) Recurrent VT	Endocardial 3.5-mm tip 4 pts Multielectrode catheters 16 pts	1) Substrate mapping: annotation of LP (=fractionated/isolated after QRS offset) and assessment if LP showed additional delay of >10 ms after RV extrastimuli (S1 600 ms, S2 VERP + 20 ms) defined as DEEP 2) PES 3) Additional mapping	1) Targeting areas with DEEP 2) Ablation of VT-related sites discretion of operator	1) Noninducibility—achieved in 80% after DEEP ablation — Remains 80% after additional ablation in those inducibLe	RF time 30.6 ± 21.4 min Procedure time and complications not reported	VT recurrence 25% at 6-month follow-up
de Riva et al. (2018) (S9.5.9) One center observational	60	33 ± 12	30%	1) Post-MI 2) Sustained VT	Endocardial Epicardial 10% — Endocardial failure — Epicardial substrate suspected 3.5-mm tip catheter	1) PES 2) Substrate mapping: systematic assessment of presumed infarct area independent of BV during SR and RV extrastimuli Pacing (S1 500 ms, S2 VRP + 50ms): EDP (evoked delayed potentials) = low voltage (<1.5 mV) EGM with conduction delay >10 ms or block in response to S2 3) Activation and pace mapping	1) Targeting EDPs only 2) Ablation of VT-related sites based on activation/pace mapping	1) Elimination of EDPs — achieved in all 2) Noninducibility of targeted VT (fast VT with VTCL≈VERP not targeted) — Achieved in 67% after EDP ablation — Achieved in 90% after additional ablation	RF time 15 min (10–21) Procedure time 173 min (150–205) Complications 3.3% One procedure-related death	VT recurrence 22% at median follow-up of 16 months (8–23) Subgroup of patients with EDPs in normal-voltage areas at baseline (hidden substrate) compared to historical matched group without EDP mapping VT-free survival at 1 year 89% vs 73%

Included studies: post myocardial infarction (or data for patients post myocardial infarction provided).

AAD = antiarrhythmic drug; BV = bipolar voltage; BZ = border zone; CABG = coronary artery bypass grafting; CE-MRI = contrast-enhanced magnetic resonance imaging; DC = delayed component; DEEP = decremental evoked potential; ECG = electrocardiogram; EDP = evoked delayed potential; EF = ejection fraction; EGM = electrogram; ICD = implantable cardioverter defibrillator; LAVA = local abnormal ventricular activity; MI = myocardial infarction; PEA = pulseless electrical activity; PES = programmed electrical stimulation; pts = patients; PVC = premature ventricular complex; RCT = randomized controlled trial; RF = radiofrequency; RFCA = radiofrequency catheter ablation; RV = right ventricle; SHD = structural heart disease; SR = sinus rhythm; VT = ventricular tachycardia.

**Table 8:** Catheter ablation of ventricular arrhythmias in cardiac sarcoidosis

Study	N	LVEF, %	Concurrent immunosuppressive therapy, n (%)	VTs induced, mean ± SD	Mapping, Endo n/Epi n	Ablation, Endo n/Epi n	Patients undergoing repeated procedures, n (%)	VT Recurrence, n (%)	VT Burden decrease, n (%)	Major complications	Follow-up, months

Koplan et al. (S9.12.5)	8	35 ± 15	5 (63)	4 ± 2	6/2	8/2	1 (13)	6 (75)	4 (44)	NR	6
Jefic et al. (S9.12.2)	9	42 ± 14	8 (89)	5 ± 7	8/1	NR	3 (33)	4 (44)	9 (100)	NR	20
Naruse et al. (S9.12.3)	14	40 ± 12	12 (86)	3 ± 1	14/0	14/0	4 (29)	6 (43)	NR	NR	33
Dechering et al. (S9.12.1)	8	36 ± 19	NR	4 ± 2	NR	NR	NR	1 (13)	7 (88)	NR	6
Kumar et al. (S9.12.6)	21	36 ± 14	12 (57)	Median 3 (range 1–8)	21/8	21/5	11 (52)	15 (71)	16 (76)	4.7%	24
Muser et al. (S9.12.4)	31	42 ± 15	22 (71)	Median 3 (range 1–5)	31/11	31/8	9 (29)	16 (52)	28 (90)	4.5%	30

LVEF = left ventricular ejection fraction; N = number; NR = not reported; VT = ventricular tachycardia.

**Table 9:** Postprocedural care in prospective studies of ventricular tachycardia catheter ablation

Study	Postprocedure NIPS	AAD type	AAD duration	Follow-up	ICD programming	Anticoagulation postablation	Bleeding and thromboembolic events (ablation arm)

Calkins 2000 (S10.1.2.5)	No	Patients were continued on the type of antiarrhythmic therapy they had received before ablation.	At least the first 3 months after hospital discharge	Evaluation at 1, 3, 6, 9, 12, and 24 months after ablation	Not specified	Not specified	Four of 146 (2.7%) stroke or TIA, 4 (2.7%) episodes of pericardial tamponade
SMASH-VT 2007 (S10.1.2.6)	No	No patient received an AAD (other than beta blockers) before the primary endpoint was reached.	N/A	Followed in the ICD clinic at 3, 6, 9, 12, 18, and 24 months; echocardiography at 3 and 12 months	Not specified	Oral anticoagulation 4–6 weeks, aspirin if fewer than 5 ablation lesions	One pericardial effusion without tamponade, managed conservatively; 1 deep venous thrombosis
Stevenson 2008 (S10.1.2.1)	No	The previously ineffective AAD was continued for the first 6 months, after which time drug therapy was left to the discretion of the investigator.	Six months, after which time drug therapy was left to the discretion of the investigator	Echocardiogram and neurologist examination before and after ablation; office visit at 2 and 6 months, with ICD interrogation where applicable	Not specified	Three months with either 325 mg/day aspirin or warfarin if ablation had been performed over an area over 3 cm in length	Vascular access complications in 4.7%; no thromboembolic complications
Euro-VT 2010 (S10.1.2.7)	No	Drug management during follow-up was at the discretion of the investigator.	Drug management during follow-up was at the discretion of the investigator.	At 2, 6, and 12 months, with ICD interrogation where applicable	Investigators were encouraged to program ICD detection for slow VT for at least 20 beats or 10 seconds to allow nonsustained VT to terminate before therapy is triggered.	Not specified	No major bleeding or thromboembolic complications
VTACH 2010 (S10.1.2.8)	No	Discouraged	Discouraged	Every 3 months from ICD implantation until completion of the study	VF zone with a cutoff rate of 200–220 bpm and a VT zone with a cutoff CL of 60 ms above the slowest documented VT and ATP followed by shock	Not specified	One transient ischemic ST-segment elevation; 1 TIA
CALYPSO 2015 (S10.1.2.9)	No	Discouraged	Discouraged	At 3 and 6 months	Investigators were required to ensure that VT detection in the ICD is programmed at least 10 beats below the rate of the slowest documented VT.	At the discretion of the treating physician, anticoagulation recommended with aspirin or warfarin for 6–12 weeks	
Marchlinski 2016 (S10.1.2.10)	Not required	Not dictated by the study protocol	Not dictated by the study protocol	At 6 months and at 1, 2, and 3 years	Not dictated by the study protocol	Per clinical conditions and physician preference	Cardiac perforation (n = 1), pericardial effusion (n = 3)
VANISH 2016 (S10.1.2.11)	No	Continued preprocedure antiarrhythmic medications	Not specified	A 3-month office visit, echo, ICD check; a 6-month office visit, ICD check; every 6 months thereafter, an office visit, ICD check	VT detection at 150 bpm or with a 10–20 bpm margin if the patient was known to have a slower VT. ATP was recommended in all zones. The protocol was modified to recommend prolonged arrhythmia detection duration for all patients.	Intravenous heparin (without bolus) 6 hours after sheath removal, then warfarin if substrate-mapping approach used or if more than 10 minutes of RF time	Major bleeding in 3 patients; vascular injury in 3 patients; cardiac perforation in 2 patients
SMS 2017 (S10.1.2.12)	No	At the discretion of the investigator	At the discretion of the investigator	At 3, 6, 9, and 12 months, and at 3- or 6-month intervals until completion of the study or until 33-month follow-up was reached	VF zone at 200–220 bpm, detection 18 of 24 beats, shock only; VT zone detection at least 16 consecutive beats, ATP, and shocks. Where VT rates were exclusively >220 bpm, VT zone at 160–180 bpm was recommended; where VT rates were <220 bpm, VT zone with a CL 60 ms above the slowest VT was recommended	Aspirin (250 mg/day) or warfarin as necessitated by the underlying heart disease	Two tamponades requiring pericardiocentesis

AAD = antiarrhythmic dug; ATP = antitachycardia pacing;CL = cycle length; ICD = implantable cardioverter defibrillator; NIPS = noninvasive programmed stimulation; RF = radiofrequency; TIA = transient ischemic attack; VF = ventricular fibrillation; VT = ventricular tachycardia.

**Table 10:** Major complications of ventricular arrhythmia ablation in patients with structural heart disease

Complication	Incidence	Mechanisms	Presentation	Prevention	Treatment	Ref.
In-hospital mortality	0%–3%	VT recurrence, heart failure, complications of catheter ablation	Not applicable	Correct electrolyte disturbances and optimize medical status before ablation	—	S10.2.1–S10.2.5
Long-term mortality	3%–35% (12–39 months of follow-up)	VT recurrence and progression of heart failure	Cardiac nonarrhythmic death (heartfailure) and VT recurrence	Identification of patients with indication for heart transplantation	—	S10.2.2–S10.2.5
Neurological complication (stroke, TIA, cerebral hemorrhage)	0%–2.7%	Emboli from left ventricle, aortic valve, or aorta; cerebral bleeding	Focal or global neurological deficits	Careful anticoagulation control; ICE can help detection of thrombus formation, and of aortic valve calcification; TEE to assess aortic arch	Thrombolytic therapy	S10.2.1–S10.2.5
Pericardial complications: cardiac tamponade, hemopericardium, pericarditis	0%–2.7%	Catheter manipulation, RF delivery, epicardial perforation	Abrupt or gradual fall in blood pressure; arterial line is recommended in ablation of complex VT	Contact force can be useful, careful in RF delivery in perivenous foci and RVOT	Pericardiocentesis; if necessary, surgical drainage, reversal heparin; steroids and colchicine in pericarditis	S10.2.1–S10.2.5
AV block	0%–1.4%	Energy delivery near the conduction system	Fall in blood pressure and ECG changes	Careful monitoring when ablation is performed near the conduction system; consider cryoablation	Pacemaker; upgrade to a biventricular pacing device might be necessary	S10.2.1–S10.2.4
Coronary artery damage/MI	0.4%–1.9%	Ablation near coronary artery, unintended coronary damage during catheter manipulation in the aortic root or crossing the aortic valve	Acute coronary syndrome; confirmation with coronary catheterization	Limit power near coronary arteries and avoid energy delivery <5 mm from coronary vessel; ICE is useful to visualize the coronary ostium	Percutaneous coronary intervention	S10.2.1–S10.2.5
Heart failure/pulmonary edema	0%–3%	External irrigation, sympathetic response due to ablation, and VT induction	Heart failure symptoms	Urinary catheter and careful attention to fluid balance and diuresis, optimize clinical status before ablation, reduce irrigation volume if possible (decrease flow rates or use closed irrigation catheters)	New/increased diuretics	S10.2.2–S10.2.5
Valvular injury	0%–0.7%	Catheter manipulation, especially retrograde crossing the aortic valve and entrapment in the mitral valve; energy delivery to subvalvular structures, including papillary muscle	Acute cardiovascular collapse, new murmurs, progressive heart failure symptoms	Careful catheter manipulation; ICE can be useful for identification of precise location of energy delivery	Echocardiography is essential in the diagnosis; medical therapy, including vasodilators and dobutamine before surgery; IABP is useful in acute mitral regurgitation and is contraindicated in aortic regurgitation	S10.2.2–S10.2.5
Acute periprocedural hemodynamic decompensation, cardiogenic shock	0%–11%	Fluid overloading, general anesthesia, sustained VT	Sustained hypotension despite optimized therapy	Close monitoring of fluid infusion and hemodynamic status -Optimize medical status before ablation -pLVAD -Substrate mapping preferred, avoid VT induction in higher-risk patients	Mechanical HS	S10.2.2–S10.2.6
Vascular injury: hematomas, pseudoaneurysm, AV fistulae	0%–6.9%	Access to femoral arterial and catheter manipulation	Groin hematomas, groin pain, fall in hemoglobin	Ultrasound-guided access	Ultrasound-guided compression, thrombin injection, and surgical closure	S10.2.1–S10.2.5
Overall major complications with SHD	3.8%–11.24%					S10.2.1–S10.2.5
Overall all complications	7%–14.7%					S10.2.3,S10.2.7,S10.2.8

AV = atrioventricular; ECG = electrocardiogram; HS = hemodynamic support; IABP = intra-aortic balloon pump; ICE = intracardiac echocardiography; MI = myocardial infarction; pLVAD = percutaneous left ventricular assist device; RF = radiofrequency; RVOT = right ventricular outflow tract; SHD = structural heart disease; TEE = transesophageal echocardiography; TIA = transient ischemic attack; VT = ventricular tachycardia.

## Appendix

**Appendix 1:** Author disclosure table

Writing group member	Employment	Honoraria/Speaking/Consulting	Speakers’ bureau	Research*	Fellowship support*	Ownership/Partnership/Principal/Majority stockholder	Stock or stock options	Intellectual property/Royalties	Other

Edmond M. Cronin, MB, BCh, BAO, FHRS, CCDS, CEPS-A (Chair)	Hartford Hospital, Hartford, Connecticut	None	None	None	None	None	None	None	None
Frank M. Bogun, MD (Vice-Chair)	University of Michigan, Ann Arbor, Michigan	None	None	None	None	None	None	None	None
Philippe Maury, MD (EHRA Chair)	University Hospital Rangueil, Toulouse, France	None	None	None	None	None	None	None	None
Petr Peichl, MD, PhD (EHRA Vice-Chair)	Institute for Clinical and Experimental Medicine, Prague, Czech Republic	1: Abbott; 1: Biosense Webster; 1: BIOTRONIK	None	None	None	None	None	None	None
Minglong Chen, MD, PhD, FHRS (APHRS Chair)	Jiangsu Province Hospital, The First Affiliated Hospital of Nanjing Medical University, Nanjing, China	None	None	None	None	None	None	None	0: APHRS Board Member
Narayanan Namboodiri, MBBS, MD (APHRS Vice-Chair)	Sree Chitra Institute for Medical Sciences and Technology, Thiruvananthapuram, India	None	None	None	None	None	None	None	None
Luis Aguinaga, MD, PhD, FESC, FACC (LAHRS Chair)	Centro Privado de Cardiología, Tucuman, Argentina	None	None	None	None	None	None	None	None
Luiz Roberto Leite, MD, PhD, FHRS (LAHRS Vice-Chair)	Instituto Brasília de Arritmia, Brasília, Brazil	None	1: Boehringer Ingelheim	None	None	None	None	None	None
Sana M. Al-Khatib, MD, MHS, FHRS, CCDS	Duke University Medical Center, Durham, North Carolina	None	None	None	None	None	None	None	None
Elad Anter, MD	Beth Israel Deaconess Medical Center, Boston, Massachusetts	2: Itamar Medical; 3: Boston Scientific; 4: Biosense Webster	None	None	None	None	None	None	None
Antonio Berruezo, MD, PhD	Heart Institute, Teknon Medical Center, Barcelona, Spain	1: Biosense Webster	1: Biosense Webster	2: Biosense Webster	None	None	None	None	None
David J. Callans, MD, FHRS, CCDS	University of Pennsylvania, Philadelphia, Pennsylvania	1: Abbott Laboratories; 1: BIOTRONIK; 1: Medtronic; 1: Wolters Kluwer; 2: Boston Scientific	None	4: NIH	2: BIOTRONIK; 2: Boston Scientific; 4: Abbott; 4: Biosense Webster; 4: Medtronic	None	None	None	1: Acutus Medical; 1: AtriCure; 1: Impulse Dynamics; 1: Thermedical; 3: Bayer
Mina K. Chung, MD, FHRS	Cleveland Clinic, Cleveland, Ohio	2: ABIM	None	5: AHA; 5: NIH	None	None	None	1: Elsevier; 1: Up to Date	0: ACC (EP Section Leadership Council member); 0: AHA (Chair, ECG & Arrhythmias Committee; Member, Clinical Cardiology Leadership Committee; Member, Committee on Scientific Sessions Programming); 0: Amarin (Data monitoring committee member); 0: BIOTRONIK; 2: AHA (Associate Editor, *Circulation Arrhythmia & Electrophysiology*)
Phillip Cuculich, MD	Washington University School of Medicine, St. Louis, Missouri	2: Medtronic	None	None	None	None	None	None	None
Andre d’Avila, MD, PhD	Hospital Cardiologico SOS Cardio, Florianopolis, Brazil	None	None	None	None	None	None	None	None
Barbara J. Deal, MD, FACC	Northwestern University Feinberg School of Medicine, Chicago, Illinois	None	None	None	None	None	None	None	None
Paolo Della Bella, MD	Ospedale San Raffaele, Milan, Italy	2: Biosense Webster; 2: Boston Scientific; 3: Abbott	None	None	2: Medtronic; 3: Abbott Vascular; 3: Biosense Webster; 3: BIOTRONIK; 4: Boston Scientific	None	None	None	None
Thomas Deneke, MD, PhD, FHRS	Herz- und Gefäß-Klinik, Bad Neustadt, Germany	None	None	None	None	None	None	None	None
Timm-Michael Dickfeld, MD, PhD, FACC, FHRS	University of Maryland, Baltimore, Maryland	1: Abbott Laboratories; 1: Biosense Webster; 1: Impulse Dynamics; 1: Philips	None	1: GE Healthcare	None	None	None	None	None
Claudio Hadid, MD	Hospital General de Agudos Cosme Argerich, Buenos Aires, Argentina	None	None	None	None	None	None	None	None
Haris M. Haqqani, MBBS, PhD, FHRS	University of Queensland, The Prince Charles Hospital, Chermside, Australia	0: Abbott Laboratories; 0: Boston Scientific; 0: Medtronic	None	3: Biosense Webster	None	None	None	None	None
G. Neal Kay, MD, CCDS	University of Alabama at Birmingham, Birmingham, Alabama	None	None	None	None	None	None	None	None
Rakesh Latchamsetty, MD, FHRS	University of Michigan, Ann Arbor, Michigan	None	1: BIOTRONIK	None	None	None	None	None	None
Francis Marchlinski, MD, FHRS	University of Pennsylvania, Philadelphia, Pennsylvania	1: Abbott; 1: Biosense Webster; 1: Boston Scientific; 1: Medtronic; 2: BIOTRONIK	None	1: Abbott; 4: Biosense Webster	2: BIOTRONIK; 2: Boston Scientific; 4: Abbott; 4: Biosense Webster; 4: Medtronic	None	None	None	None
John M. Miller, MD, FHRS	Indiana University School of Medicine, Krannert Institute of Cardiology, Indianapolis, Indiana	1: Abbott Laboratories; 1: Biosense Webster; 1: Boston Scientific; 1: BIOTRONIK; 1: Medtronic	None	None	3: Biosense Webster; 3: Boston Scientific; 3: Medtronic	None	None	1: Elsevier	None
Akihiko Nogami, MD, PhD	University of Tsukuba, Ibaraki, Japan	1: Abbott Laboratories	None	4: Medtronic	None	None	None	None	None
Akash R. Patel, MD, FHRS, CEPS-P	University of California San Francisco Benioff Children’s Hospital, San Francisco, California	None	None	None	None	None	None	None	None
Rajeev Kumar Pathak, MBBS, PhD, FHRS	Australian National University, Canberra Hospital, Canberra, Australia	1: BIOTRONIK	1: Medtronic	None	None	None	None	None	None
Luis C. Saenz Morales, MD	CardioInfantil Foundation, Cardiac Institute, Bogota, Columbia	None	None	None	None	None	None	None	None
Pasquale Santangeli, MD, PhD	University of Pennsylvania, Philadelphia, Pennsylvania	1: Abiome; 1: Baylis Medical; 1: Biosense Webster; 1: Medtronic; 1: Stereotaxis; 2: Abbott Laboratories	None	None	None	None	None	None	None
John L. Sapp, Jr., MD, FHRS	Queen Elizabeth II Health Sciences Centre, Halifax, Canada	1: Abbott Vascular; 1: Biosense Webster; 1: Medtronic	None	4: Abbott Vascular; 5: Biosense Webster	None	None	None	1: Biosense Webster	None
Andrea Sarkozy, MD, PhD, FEHRA	University Hospital Antwerp, University of Antwerp, Antwerp, Belgium	1: Biosense Webster; 1: BIOTRONIK	None	None	None	None	None	None	0: EHRA Board member
Kyoko Soejima, MD	Kyorin University School of Medicine, Tokyo, Japan	2: Abbott Laboratories; 2: Boehringer Ingelheim	3: Medtronic	None	None	None	None	None	None
William G. Stevenson, MD, FHRS	Vanderbilt University Heart and Vascular Center, Nashville, Tennessee	1: Abbott; 1: BIOTRONIK; 1: Boston Scientific; 1: Medtronic	None	None	None	None	None	0: Biosense Webster; 0: Brigham and Women’s Hospital	None
Usha B. Tedrow, MD, MS, FHRS	Brigham and Women’s Hospital, Boston, Massachusetts	1: Abbott; 1: Biosense Webster; 1: Medtronic	None	None	None	None	None	None	None
Wendy S. Tzou, MD, FHRS	University of Colorado Denver, Aurora, Colorado	1: Abbott; 1: Biosense Webster; 1: BIOTRONIK; 1: Boston Scientific; 1: Medtronic	None	None	None	None	None	None	None
Niraj Varma, MD, PhD	Cleveland Clinic, Cleveland, Ohio	1: BIOTRONIK; 1: Medtronic	None	3: Abbott	None	None	None	None	None
Katja Zeppenfeld, MD, PhD, FESC, FEHRA	Leiden University Medical Center, Leiden, the Netherlands	1: Abbott	None	5: Biosense Webster	3: Biosense Webster	None	None	None	None

Number value: **0** = $0; **1** = ≤ $10,000; **2** = > $10,000 to ≤ $25,000; **3** = > $25,000 to ≤ $50,000; **4** = > $50,000 to ≤ $100,000; **5** = > $100,000.

ABIM = American Board of Internal Medicine; ACC = American College of Cardiology; AHA = American Heart Association; APHRS = Asia Pacific Heart Rhythm Society; EHRA = European Heart Rhythm Association; LAHRS = Latin American Heart Rhythm Society; NIH = National Institutes of Health.

* Research and fellowship support are classed as programmatic support. Sources of programmatic support are disclosed but are not regarded as a relevant relationship with industry for writing group members or reviewers.

**Appendix 2:** Reviewer disclosure table

Peer reviewer	Employment	Honoraria/Speaking/Consulting	Speakers’ bureau	Research*	Fellowship support*	Ownership/Partnership/Principal/Majority stockholder	Stock or stock options	Intellectual property/Royalties	Other

Samuel J. Asirvatham, MD, FHRS	Mayo Clinic College of Medicine, Rochester, Minnesota	1: Abbott; 1: BIOTRONIK; 1: Boston Scientific; 1: Medtronic	None	None	None	None	None	1: AliveCor	None
Eduardo Back Sternick, MD, PhD	Faculdade Ciências Médicas de Minas, Gerais, Brazil	None	None	None	None	None	None	None	None
Janice Chyou, MD	Northport VA Medical Center, Northport, New York; Agile Health, New York, New York	None	None	None	None	None	None	None	None
Sabine Ernst, MD, PhD	Royal Brompton and Harefield Hospitals, London, England	2: Biosense Webster; 2: Stereotaxis	None	3: Other; 4: Baylis; 4: Spectrum Dynamics	None	None	None	None	None
Guilherme Fenelon, MD, PhD	Hospital Israelita Albert Einstein, Sao Paulo, Sao Paulo, Brazil	1: Libbs	None	None	None	None	None	None	None
Edward P. Gerstenfeld, MD, MS, FACC	University of California, San Francisco, San Francisco, California	1: Abbott Vascular; 1: Biosense Webster; 1: Boston Scientific; 1: Medtronic	None	4: Abbott Vascular; 4: Biosense Webster	None	None	None	None	None
Gerhard Hindricks, MD	Heart Center Leipzig at the University of Leipzig, Leipzig, Germany	None	None	4: Abbott Vascular	None	None	None	None	None
Koichi Inoue, MD, PhD	Sakurabashi-Watanabe Hospital, Osaka, Japan	2: Biosense Webster; 2: Medtronic Japan	None	None	None	None	None	None	None
Jeffrey J. Kim, MD	Baylor College of Medicine, Texas Children’s Hospital, Houston, Texas	None	None	None	3: Medtronic	None	None	None	None
Kousik Krishnan, MD, FHRS, FACC	Rush University Medical Center, Chicago, Illinois	1: ZOLL	None	None	None	None	None	None	None
Karl-Heinz Kuck, MD, FHRS	Asklepios Klinik St. Georg, Hamburg, Germany	1: Abbott Vascular; 1: Biosense Webster; 1: Boston Scientific; 1: Edwards Lifesciences; 1: Medtronic	None	None	None	None	None	None	None
Martin Ortiz Avalos, MD	Hospital San Angel Inn Universidad, Mexico City, Mexico	None	None	None	None	None	None	None	None
Thomas Paul, MD, FACC, FHRS	Georg August University Medical Center, Gottingen, Germany	None	None	None	None	None	None	None	None
Mauricio I. Scanavacca, MD, PhD	Instituto Do Coracao, Sao Paulo, Brazil	None	None	None	None	None	None	None	None
Roderick Tung, MD, FHRS	The University of Chicago Medicine, Center for Arrhythmia Care, Heart and Vascular Center, Chicago, Illinois	2: Abbott	None	2: Abbott	3: Abbott; 3: Medtronic; 3: Boston Scientific	None	None	None	None
Jamie Voss, MBChB	Middlemore Hospital, Auckland, New Zealand	None	None	None	None	None	None	None	None
Takumi Yamada, MD	University of Alabama at Birmingham, Birmingham, Alabama	1: Nihon Kohden; 2: Abbott; 2: Japan Lifeline	None	None	None	None	None	None	None
TeiichiYamane, MD, PhD, FHRS	Jikei University School of Medicine, Tokyo, Japan	1: Boehringer Ingelheim; 1: Bristol-Myers Squibb; 2: Abbott Laboratories; 2: Medtronic Japan	None	None	None	None	None	None	None

Number value: 0 = $0; 1 = ≤ $10,000; 2 = > $10,000 to ≤ $25,000; 3 = > $25,000 to ≤ $50,000; 4 = > $50,000 to ≤ $100,000; 5 = > $100,000.

* Research and fellowship support are classed as programmatic support. Sources of programmatic support are disclosed but are not regarded as a relevant relationship with industry for writing group members or reviewers.

## ABBREVIATIONS

AAD: antiarrhythmic drug
AIV: anterior interventricular vein
AMC: aortomitral continuity
ARVC: arrhythmogenic right ventricular cardiomyopathy
ATP: antitachycardia pacing
AV: atrioventricular
BBRVT: bundle branch reentrant ventricular tachycardia
CHD: congenital heart disease
CMR: cardiac magnetic resonance imaging
COR: class of recommendation
CS: coronary sinus
DCM: dilated cardiomyopathy
EAM: electroanatomical mapping
ECG: electrocardiogram
GCV: great cardiac vein
HCM: hypertrophic cardiomyopathy
HS: hemodynamic support
ICD: implantable cardioverter defibrillator
ICE: intracardiac echocardiography
ICM: ischemic cardiomyopathy
IHD: ischemic heart disease
LBB: left bundle branch
LBBB: left bundle branch block
LMNA: lamin A/C
LOE: level of evidence
LSV: left sinus of Valsalva
LV: left ventricle
LVOT: left ventricular outflow tract
NCSV: noncoronary sinus of Valsalva
NICM: nonischemic cardiomyopathy
PES: programmed electrical stimulation
PVC: premature ventricular complex
RBB: right bundle branch
RBBB: right bundle branch block
RSV: right sinus of Valsalva
RV: right ventricle
RVOT: right ventricular outflow tract
RWI: relationship with industry and other entities
SHD: structural heart disease
SV: sinus of Valsalva
VA: ventricular arrhythmia
VF: ventricular fibrillation
VT: ventricular tachycardia (Heart Rhythm 2020;17:e155–e205)
